# Meta-Prediction of *MTHFR* Gene Polymorphisms and Air Pollution on the Risk of Hypertensive Disorders in Pregnancy Worldwide

**DOI:** 10.3390/ijerph15020326

**Published:** 2018-02-13

**Authors:** Ya-Ling Yang, Hsiao-Ling Yang, S. Pamela K. Shiao

**Affiliations:** 1 School of Nursing, College of Medicine, National Taiwan University, Taipei 10051, Taiwan; ylyang@ntu.edu.tw (Y.-L.Y.); slyang@ntu.edu.tw (H.-L.Y.); 2 College of Nursing, Augusta University, Augusta, GA 30912, USA

**Keywords:** *methylenetetrahydrofolate reductase*, polymorphism, hypertensive disorder in pregnancy, air pollution, meta-predictive analysis

## Abstract

Hypertensive disorders in pregnancy (HDP) are devastating health hazards for both women and children. Both *methylenetetrahydrofolate reductase* (*MTHFR*) gene polymorphisms and air pollution can affect health status and result in increased risk of HDP for women. The major objective of this study was to investigate the effect of *MTHFR* polymorphisms, air pollution, and their interaction on the risk of HDP by using meta-predictive analytics. We searched various databases comprehensively to access all available studies conducted for various ethnic populations from countries worldwide, from 1997 to 2017. Seventy-one studies with 8064 cases and 13,232 controls for *MTHFR C677T* and 11 studies with 1425 cases and 1859 controls for *MTHFR A1298C* were included. *MTHFR C677T homozygous TT* (risk ratio (RR) = 1.28, *p* < 0.0001) and *CT plus TT* (RR = 1.07, *p* = 0.0002) were the risk genotypes, while *wild-type CC* played a protective role (RR = 0.94, *p* = 0.0017) for HDP. The meta-predictive analysis found that the percentage of *MTHFR C677T TT plus CT* (*p* = 0.044) and *CT* (*p* = 0.043) genotypes in the HDP case group were significantly increased with elevated levels of air pollution worldwide. Additionally, in countries with higher air pollution levels, the pregnant women with *wild-type CC MTHFR 677* had a protection effect against HDP (*p* = 0.014), whereas, the *homozygous TT* of *MTHFR C677T* polymorphism was a risk genotype for developing HDP. Air pollution level is an environmental factor interacting with increased *MTHFR C677T* polymorphisms, impacting the susceptibility of HDP for women.

## 1. Introduction

Hypertensive disorders in pregnancy (HDP) are major health hazards in perinatal care [1,2]. Women with HDP, with a prevalence rate of 5.2~8.2% of all pregnancies [3], have 1.4 to 3-fold increased risk of developing cardiovascular disease (CVD) later in life [3,4,5,6], and 14% chance of maternal deaths [4]. HDP increases the risk of premature births 80-fold and causes 3 to 12% of perinatal mortality in the western countries [7,8]. However, the etiology of HDP remains unclear, though known risk factors may stem from genetics, the environment, and their interactions [8,9,10].

The *methylenetetrahydrofolate reductase* (*MTHFR*) gene has been reported as a candidate gene associated with HDP [11,12,13]. MTHFR, an important enzyme, catalyzes 5,10-methylenetetra- hydrofolate to 5-methyltetrahydrofolate, which serves as a methyl donor for homocysteine re- methylation for DNA synthesis and repair [14]. Two common *MTHFR* polymorphism-mutation loci include *C677T* (rs1801133) and *A1298C* (rs1801131), both are associated with MTHFR enzymatic deficiency resulting in increased homocysteine concentrations [13,14]. Hyperhomocysteinemia may lead to micro- and macro-vascular thrombosis, which impairs endothelial trophoblast growth and differentiation [15,16,17] and placental dysfunction, aberrant endothelial function [14], atherosclerosis, HDP [18], and the HELLP syndrome (hemolysis, elevated liver enzymes, and low platelet count) [19]. Reduced MTHFR enzyme activity can also alter genome instability with free radicals causing accumulation of toxins [14]. To date, numerous epidemiological studies and meta-analyses [9,20,21,22] confirmed that *MTHFR C677T* [10,17,23,24] and *MTHFR A1298C* [13,24,25] polymorphisms are associated with HDP; however, with inconsistent findings [26,27,28].

Further evidence has indicated that HDP can result from epigenetic factors, as well as modifications and interactions involving genetic factors and environmental toxicants [29,30]. Exposure to air polluted by fine particulate matter (PM_2.5_) [26,27], ozone (O_3_), carbon monoxide (CO), nitrogen oxides (NOx), and nitrogen dioxide (NO2) [29,31,32,33,34,35,36] can trigger inflammatory reactions, increase blood coagulation, and decrease placental circulation. Consequently, these toxicants can lead to HDP [30,37], though the underlying mechanisms remain unclear [29,35]. Further evidence indicates that *MTHFR C677T* polymorphism, *CT* or *TT* genotypes, and exposure to air pollution may significantly increase the risk of CVD across the lifespan [38].

In summary, previous meta-analyses examined the association between *MTHFR C677T* and *A1298C* polymorphisms with HDP [9,20,21,22] and presented that *MTHFR C677T* polymorphism had significantly increased the susceptibility of HDP for various ethnic groups. However, none of these meta-analyses addressed the effects of epigenetic factors including air pollution on the development of HDP. Therefore, the primary objective of this study is to examine the *MTHFR* gene polymorphism on HDP risk across the globe. In addition, the secondary objective is to investigate the impact of air pollution on *MTHFR* polymorphisms and the risks of HDP, using meta-predictive techniques.

## 2. Materials and Methods

We conducted a comprehensive literature search, following the guidelines of meta-analysis of observational studies in epidemiology (MOOSE) [39] and preferred reporting items for systematic reviews and meta-analyses (PRISMA) statement [40] for reporting items in this meta-analysis.

### 2.1. Study Search Strategy

We searched various online databases of PubMed, PubMed Central, Embase, and Airiti Library to access all available studies from the first relevant study in 1997 to 2017. We used combinations of the following keywords: “*MTHFR* gene” or “*MTHFR* polymorphism” or “*methylenetetrahydrofolate reductase* gene” or “*MTHFR* in pregnant women”, and mash terms for “new-onset hypertensive disorders during pregnancy” [1,2] including “pregnancy induced hypertension” or “preeclampsia (PE)” or “eclampsia” or “hypertension in/during pregnancy” or “gestational hypertension (GH)” or “pregnancy complication”, and “meta-analysis” or “case-control” or “case control design” for studies including the genotype allele counts for both cases and controls. In this study, we focused on the new-onset hypertension during pregnancy, excluding preexisting hypertension in the classification of HDP [1,2]. We searched the various databases thoroughly at three different times at least 3 months apart from 2014 to 2017 until we could not identify additional papers. In addition, we used previous meta-analysis and review papers to trace back to all original studies. Two investigators, both familiar with the literature-search process and organization and one familiar with meta-analytic methods, conducted the literature search to identify all possible original studies.

### 2.2. Selection Criteria and Study Identification

**Figure 1:**
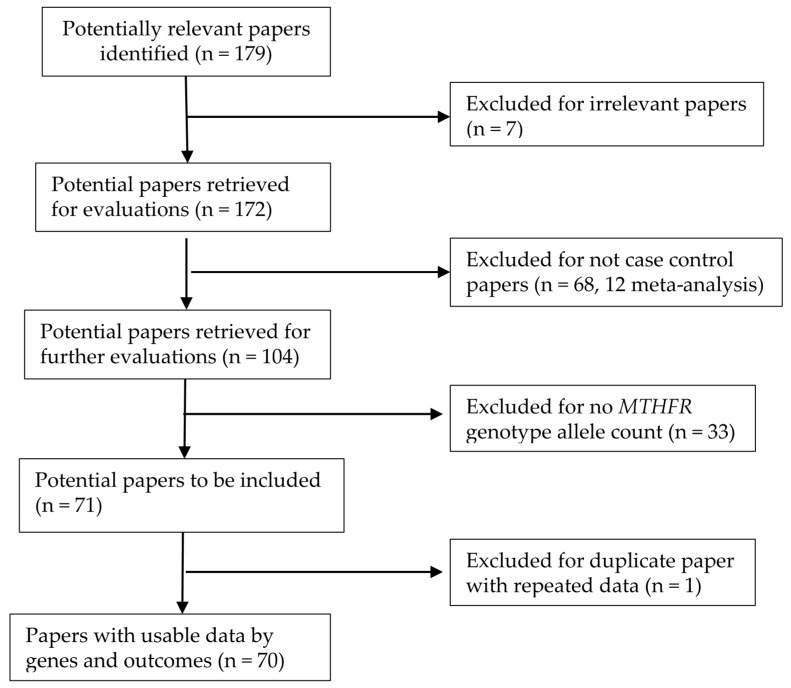
Progression on the selection of papers for the meta-analysis.

We selected articles that examined the association between *MTHFR C677T* and *MTHFR A1298C* polymorphisms and HDP and that clearly reported appropriate genotype allele counts per case and control groups. We included studies if the articles included an abstract in English and tables that clearly listed the genotype allele counts. We excluded articles that (1) did not include genotypes per case and control groups and (2) did not include genotyping in pregnant women. Figure 1 presents our study selection process. Originally, we located 179 articles that were related to *MTHFR C677T* or *MTHFR A1298C* polymorphisms and HDP. We excluded a total of 109 articles, including 7 irrelevant studies, 68 none-case-control studies, 33 studies with missing *MTHFR* genotype allele counts, and 1 study involving subsidiary data repeated in another study (Table S1). Consequently, we included 70 articles with usable genotype allele count data (Figure 1). One article [41] included three racial-ethnic groups (Germany, Croatia, and Indonesia) yielding two additional study groups. Among these 72 studies, 71 studies contained data on *MTHFR C677T* genotypes and one study included only *MTHFR A1298C* genotype data. This one study, together with another 10 studies including data on both *MTHFR C677T* and *MTHFR A1298C* polymorphisms, yielded 11 studies having *MTHFR A1298C* data.

### 2.3. Characteristics of Included Studies

Study populations were drawn from Australia, Europe, North America, South America, Asia, the Middle East, and Africa. One study examined three racial-ethnic groups, and the most studied populations were Caucasian (27 studies), followed by Asian (22 studies including 18 East Asian (1 study included only *MTHFR A1298C* data), and 4 South Asian), then Hispanic (7 studies, as reported in the studies), Middle Eastern (7 studies), African (5 studies), and South American (4 studies) (Table S1). For HDP subtypes, we categorized all studies into three types of HDP: PE—eclampsia with or without HELLP syndrome (57 studies), GH (4 studies), and mixed (both GH and PE-eclampsia) subtypes (10 studies) (Table S1).

We entered the air-quality data for various countries. Specifically, we verified from various sources for the most current and complete air-pollution data including the death rates from air pollution (death rates per million, Level 1: ≤50, Level 2: 51–100, Level 3: 101–250, Level 4: 251–400, Level 5: ≥401) [42,43]. The air pollution markers we used are per country data, because the source data reported are from countries across the studies. We further verified these levels with current scales on air pollution data [44,45,46,47], and the most complete and current data on air pollution data were used for the analyses. There was only one study (Ireland) with a Level 1 pollution level and no study with Level 5; therefore, Level 1 was merged with Level 2 for final analysis.

### 2.4. Quality Assessment

We evaluated the quality of each study using a set of indicators appropriate for the current state of science for the field [48,49,50] (Table S1). The quality score of all included studies ranged from 15 to 26, greater than 50% of total possible scores (0–28) (Table S1), suggesting the finding of the studies were trustworthy [40]. We checked the Hardy-Weinberg Equilibrium (HWE) analysis to assess the distribution equilibrium of the evolutionary mechanisms in population genetics [51], associated with factors such as population migration or stratification and disease association. We performed subgroup analysis based on the HWE status, and the results showed no significant differences based on HWE status for pooled analyses. Therefore, we included all eligible studies in the final analysis, consistent with recent meta-analyses in the field [9,48]. We ran inter-rater reliability on data entry and analyses to ensure that data extraction and coding were accurate for all studies. We cross checked the data and discussed the discrepancies to reach 100% agreement among team members.

### 2.5. Data Synthesis and Analysis

We entered data into Excel spreadsheets and used StatsDirect, version 2.4.7 (Cheshire, UK) to pool data analyses. We calculated pooled relative risk ratios (RR), odds ratios (OR), and 95% confidence intervals (CI) between cases and controls for the associations of *MTHFR* polymorphism genotypes with HDP. We defined significant findings as those with *p*-values < 0.05. The results of RRs and ORs were similar (Table S2a,b); however, RRs were more robust and conservative when predicting the risks. For standardized risk, a pooled RR is preferred and has been used in most recent international consensus reports [52,53,54]. Additionally, we used standardized ratios for RRs and ORs using the total count as the denominator for all three genotypes (homozygous CC, heterozygous TC, and wild-type TT genotypes) to depict the standardized RRs (vs. use of only one of the genotypes as denominator) to further understand the sources of heterogeneity of the findings [53]. For standardized risk ratios, a pooled RR is preferred as the standardized ratio and has been used in most recent consensus reports [54]; however, as most of the previous meta-analysis reports on this topic presented ORs, they were also pooled to check the differences on the results with RRs. Furthermore, we examined the heterogeneity of results for the pooled analyses by RR of 1 (>1 or <1) as well as the sources of heterogeneity, including geographic regions and additional potential contributing factors such as air pollution levels, various HDP types, sources of control, and quality score.

The Heterogeneity tests, Egger’s test, and funnel plots were used to detect publication bias. No significant publication bias was found on the meta-analyses of *MTHFR* polymorphism tests (T = −0.1636–0.2, bias = −0.661–1.0592, all *p* > 0.05). Random effects instead of fixed effects models were used for the risk estimates when the heterogeneity tests were significant with *p* < 0.05.

Because the data showed heterogeneity with regional differences on *MTHFR* poly-morphisms and risks, we used geographic-information-system (GIS) maps to visualize the pattern distributions of polymorphisms and risks on the global maps [55]. In addition, we used recursive partition trees in the JMP 13 program (SAS Institute, Cary, NC, USA) to examine how an independent variable (the death rate from air pollution) can make a decisive split of the data: these trees partitioned the original groups into pairs of subgroups in relation to the dependent variable (polymorphism rates and risks) [56]. To judge the goodness of the partition, we used Akaike’s information criterion correction (AICc). Both GIS maps and recursive trees are common big-data analytic techniques for handling large-scale and multidimensional datasets. We applied meta-predictive analytical techniques using recursive partition tree, nonlinear fit, and heat maps for data visualization to reveal nonlinear patterns in this study, in addition to the conventional pooled-analysis technique, to visualize the heterogeneity. While meta-regression is used commonly for advanced meta-analysis for meta-prediction [54], it is important to point out that regression analysis, as a linear model, is unable to detect nonlinear patterns. Further, it is well known that regression based on *R*^2^ tends to yield a complex and overfitted model because *R*^2^ always goes up with additional predictors. On the other hand, Akaike’s information criterion (AIC) or AICc does not necessarily change with the addition of variables. Rather, it varies based upon the composition of the predictors; thus, it is more likely to yield an optimal model [57]. Furthermore, we employed a conventional multiple comparison procedure in addition to the partition tree analyses to examine whether partition trees and Tukey’s tests concurred with each other. We also used non-linear fit to examine the associations between air pollution and the outcome variables (polymorphism rates and risks). Further insights can be unveiled when the scatterplot of bivariate distributions is converted into a heat-map, in which the color spectrum represents the frequency counts [58]. These meta-analytic techniques aim to generate predictions that are more precise by integrating data from diverse sources.

## 3. Results

### 3.1. Pooled Meta-Analysis

#### 3.1.1. *MTHFR C677T*

For pooled analysis of *MTHFR C677T* polymorphisms, we analyzed 8064 HDP cases and 13,232 controls in 71 study groups for populations from all continents worldwide. For clarity, we summarized significant findings in a schema Table (Table 1) for overall populations and specific ethnic populations, as well as HDP subtypes including PE-eclampsia, GH, and mixed (both GH and PE-eclampsia). Specifically, *MTHFR C677T* polymorphism homozygous *TT* and *TT plus CT* (where both TT and CT types were added together to see the dominant effects of polymorphisms) were risk genotypes for overall populations and for the PE-eclampsia subgroup (Table 1).

For the HDP group, percent *MTHFR C677T* homozygous *TT* genotype for the total population was 13.48% (*n* = 1087), and its frequency in ethnic groups in rank order was Hispanic (28.10%), East Asian (23.59%), South American (16.40%), Caucasian (11.65%), Middle Eastern (7.26%), African (2.81%), and South Asian (2.67%) (Table 2). *Homozygous TT* genotype was a risk type of HDP for all populations combined (RR = 1.28, *p* < 0.0001); and the rank order of highest risk to lowest was African (RR = 5.82), East Asian (RR = 1.75), South American (RR = 1.40), and Caucasian (RR = 1.14) (Table 1 and Table 2). The protective genotypes were *wild-type CC* (RR = 0.94, *p* = 0.0017) for all populations combined, as well as for HDP subtypes of PE-eclampsia and GH (Table 1 and Table 2). For ethnic subgroups, *wild-type CC* was a protective genotype for East Asian, Caucasian, and African (Table 2). While *Heterozygous CT* genotype was a significant protective genotype of HDP for South Asian (*p* < 0.05); its effects were mixed without significance for all other populations (Table 2).

#### 3.1.2. MTHFR A1298C

For *MTHFR A1298C* polymorphisms, we included 1425 cases and 1859 controls from 11 study groups (Table S3). The frequency of homozygous *CC* risk genotype in HDP case group, in rank order, was South American (15.33%), Caucasian (8.96%), Middle Eastern (7.02%), Asian (6.99%), and finally African (2.04%) (Table S3). Pooled analysis did not show significant association between *MTHFR A1298C* polymorphism and HDP, except that AA wild-type was a protective genotype in the Middle Eastern populations (RR = 0.87, *p* = 0.04).

### 3.2. Subgroup Analyses by Countries and Regions

We further present the distributions of *MTHFR C677T* polymorphisms (Figure S1a) and *MTHFR A1298C* polymorphism (Figure S1b) per countries for control and HDP cases groups for the distributions of highest to lowest prevalence in various countries. We generated GIS maps and used the spectrum from yellow to red to represent the increasing percentage of *MTHFR C677T* polymorphisms *TT plus CT* genotypes (Figure S2a); and red-to-green spectrum to present disease risks, with red color indicating higher HDP risk and green indicating protective effects. Additionally, we pooled the countries with homozygous *TT* as a risk genotype (RR > 1 in 21 countries) versus those that had it as a protective genotype (RR < 1 in 5 countries) and other countries that had varied effect (RR varied around 1 in 4 countries) (Table 2; Figure S3a–c). The countries with homozygous *TT* as a risk genotype (RRs > 1) were found in 49 studies from the regions of Europe, Americas, Asia, the Middle East, and Africa (RR = 1.49, *p* < 0.0001) (Table 2 and Figure S3a). In contrast, countries with *TT* as a protective genotype (RR < 1) included Australia, Germany, Austria, Croatia, and India (Table 2 and Figure S3b). There were no significant findings on subgroup analyses for the associations between *MTHFR A1298C* polymorphism and HDP (Table S3, Figures S2b and S4a,b).

### 3.3. Subgroup-Analysis by HDP Disease Types

Subgroup-analysis by HDP disease types with *MTHFR C677T* polymorphisms demonstrated that the *homozygous TT* (RR = 1.29, *p* = 0.0001) and *TT plus CT* (RR = 1. 04, *p* = 0.0287) were risk genotypes for the PE-eclampsia subgroup. *Homozygous TT* was also a risk genotype (RR = 1.65, *p* = 0.0023) for the GH subtype. The *heterozygous CT* (RR = 1.22, *p* = 0.0162) and *TT plus CT* (RR = 1.21, *p* < 0.0001) were risk genotypes for the mixed HDP subgroup. The *wild-type CC* was a protective genotype for the PE-eclampsia, GH, and mixed HDP groups.

The *CC plus CT* polymorphisms were also protective for PE-eclampsia and GH HDP groups (Table S4a). Further analyses by ethnicity showed that *homozygous TT* was a significant risk genotype of PE-eclampsia for Caucasian, South American, East Asian, and African. In PE-eclampsia, *heterozygous CT* was a protective genotype for East and South Asian, the *wild-type CC* was protective for East Asian, and *CC plus CT* was protective for East Asian and South American. *Homozygous TT* was a significant risk genotype, and *CC plus CT* were protective for East Asian with GH. While *heterozygous CT* genotype was protective against HDP in Caucasian of the mixed HDP subgroup, *TT plus CT* subtypes were a risk in developing HDP, whereas *CC wild-type* was a protective genotype for Caucasian and East Asian (Table S4a).

For *MTHFR A1298C* polymorphisms, the most common HDP disease type was PE-eclampsia (9 studies, 1022 cases, and 1421 controls). There was no significant association between *MTHFR A1298C* genotypes and HDP risk, except *CC plus AC* genotypes were a risk and *AA wild-type* was a protective genotype for PE-eclampsia subgroup in the Middle East (Table S4b).

### 3.4. Meta-Prediction: MTHFR Polymorphisms and Air Pollution Associated with Risk of HDP

**Figure 2:**
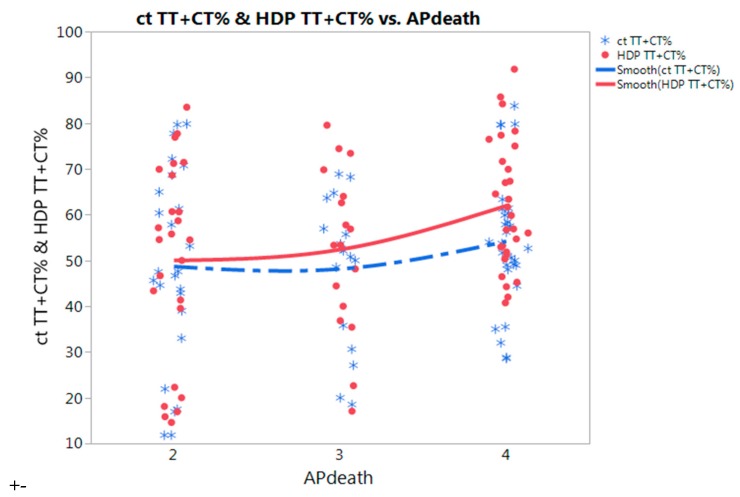
Nonlinear fit of *MTHFR* 677 *TT + CT* % polymorphism for control and hypertensive disorders in pregnancy (HDP) in association with death from air pollution (AP death: Death rates from air pollution, Levels per million: 2: ≤100, 3: 101–250, 4: >251.

Because findings were mixed on the effects of *MTHFR* polymorphisms, we performed meta-predictive analyses using both big-data analytics and conventional analyses for the risk of HDP (Table 3). We used both partition tree and Tukey’s tests to examine the impact of the interaction between air pollution and polymorphisms on HDP risk. In the HDP case group, the percentages of *TT plus CT* (*p* = 0.044) and *heterozygous CT* (*p* = 0.043) genotypes were significantly higher in countries with higher (Level 4) air pollution than in countries with lower (Level 2) air pollution, while the percentage of *wild-type CC* genotype showed reversed findings to that of other two polymorphic genotypes combined in association with the levels of air pollution exposure (*p* = 0.044) (Table 3). Interestingly, these associations were not observed in the control group (*p* > 0.05) while there was an upward trend of polymorphic genotypes. To further illustrate these significant findings, we plotted those results on nonlinear curves (Figure 2). With increasing air pollution from lower to higher levels, the percentages of *TT plus CT* genotypes showed a steeper increase in the HDP group than in the control group (Figure 2). Contrarily, *CC wild-genotype* showed higher protective effects in countries with level 4 than those in level 2 air pollution (Table 3). The heat maps illustrate data density; red blocks depict higher concentrations, data were denser for countries with higher or Level 4 air pollution (Figure S5a,b).

## 4. Discussion

Based on the results of this comprehensive analysis, we show a significant association between *MTHFR C677T* polymorphism and HDP risk for populations worldwide [9,20,21,22]. For ethnic subgroup-analysis, we found significant associations between *homozygous TT* as well as *TT plus CT* genotypes and HDP risk in East Asian [9,20,21], Caucasian [9,20,21], and African groups. Further, the *homozygous TT* genotype was found to be a risk factor for HDP in the PE-eclampsia subgroup in African [13] and South American populations [24]. We added newly published studies, and separated Latino into Hispanic and South American to increase geographic specificity.

Our pooled analyses provided summative evidence with the findings from individual studies on the direct effects of exposure to air pollution being associated with increased risk of HDP [30,33,35,36]. We applied meta-predictive analytical techniques using recursive partition tree, nonlinear fit, and heat maps for data visualization to reveal nonlinear patterns in this study, in addition to the conventional pooled-analysis technique, to visualize the heterogeneity. While meta-regression is used commonly for advanced meta-analysis for meta-prediction [54], it is important to point out that regression analysis, as a linear model, is unable to detect nonlinear patterns. AIC or AICc does not necessarily change with the addition of variables. Rather, it varies based upon the composition of the predictors; thus, it is more likely to yield an optimal model [57]. Out of many potential predictors (ethnic groups, types of cases, sources of controls, quality score), only air pollution could decisively demarcate the polymorphism-mutation and risk outcomes. Therefore, we focused our meta-prediction analysis on different air pollution levels as measured by the deaths from air pollution for the completeness of available data to all countries included in the analysis with the years of studies.

With this meta-prediction study, we found that air pollution was associated with increased *MTHFR 677* polymorphism in HDP cases and thus HDP risks and that air pollution had an additive effect to *MTHFR C677T* polymorphism in increasing the risk of HDP. Noticeably, both the nonlinear plots and heat-maps clearly show that the *MTHFR 677* polymorphisms significantly increased in the HDP case group from the countries with lower to higher levels of air pollution, but not in the control group. Previous studies presented that fine particles from air pollution can affect endothelial dysfunction causing abnormal placentation [33,35,36] and systemic inflammation during pregnancy [35,59], resulting in HDP and cardiovascular squeals. Moreover, fine particles from air pollution may cross the placenta and cause hypoxia in the fetus [36]. Continuing from the findings in previous studies, we further demonstrated the risk of CVDs during pregnancy for women are increased from both genetics and environmental factors [29,30], as HDP is related to CVD development for women across the lifespan.

## 5. Conclusions

Overall, this meta-prediction study provided a comprehensive analysis of *MTHFR* polymorphisms on the risk of HDP with the effects of air pollution in pregnant populations worldwide. Our study demonstrated the adverse effect of air pollution with increased *MTHFR C677T* polymorphisms and women’s susceptibility to HDP. Further studies are needed to examine whether and how interventions such as diets rich in folate, vitamin B6, or vitamin B12 can counteract the effects of air pollution [59] on HDP and the interaction with maternal age and histories. Further studies are warranted to examine the effects of air pollution and gene-environment interaction on maternal and fetal outcomes. Our findings may also have significant implications in advocating for public health policy changes for a clean-air environment that would promote the health of mothers and children.

## Figures and Tables

**Table 1:** Schema of significant findings across studies on methylenetetrahydrofolate reductase (*MTHFR) C677T* genotypes and the risk of hypertensive disorders in pregnancy (HDP) between cases and controls.

HDP Types	ALL	PE-E	GH	Mixed
Number of Studies	71 Studies	57 Studies	4 Studies	10 Studies
(*n* Case/*n* Control)	(8064/13,232)	(5873/11,545)	(336/327)	(1855/1360)
Overall (71 Studies)	Risk Type: *TT* and *TT*+*CT* Protective: *CC* and *CC*+*CT*	Risk Type: *TT* and *TT*+*CT* Protective: *CC* and *CC*+*CT*	Risk Type: *TT* Protective: *CC* and *CC*+*CT*	Risk Type: *CT* and *TT*+*CT* Protective: *CC*
Subgroups				
Caucasian	27 Studies (3648/7138) Risk Type: *TT* and *TT*+*CT* Protective: *CC*	25 Studies (2818/6860) Risk Type: *TT*	--	2 Studies (830/278) Risk Type: *CT* and *TT*+*CT* Protective: *CC*
Hispanic	7 Studies (765/1115) NS	6 Studies (577/921) NS	--	1 Study (188/194)
South American	4 Studies (378/555) Risk Type: *TT* Protective: *CC* and *CC*+*CT*	4 Studies (378/1255) Risk Type: *TT*	--	--
East Asian	17 Studies (1255/2030) Risk Type: *TT* and *TT*+*CT* Protective: *CC* and *CC*+*CT*	8 Studies (531/2177) Risk Type: *TT* and *TT*+*CT* Protective: *CT*, *CC* and *CC*+*CT*	3 Studies (236/225) Risk Type: *TT* Protective: *CC*+*CT*	6 Studies (488/550) Risk Type: *TT*+*CT* Protective: *CC*
South Asian	4 Studies (561/991) Protective: *CT*	4 Studies (561/991) Protective: *CT*	--	--
Middle East	7 Studies (744/628) NS	6 Studies (644/526) NS	1 Study (100/102)	--
African	5 Studies (713/775) Risk Type: *TT* and *TT*+*CT* Protective: *CC* and *CC*+*CT*	4 Studies (364/874) Risk Type: *TT*	--	1 Study (349/338)

Note: NS: Not significant; --: No data; PE-E: preeclampsia–eclampsia; GH: gestational hypertension; Mixed: PE-eclampsia and GH.

**Table 2:** Pooled meta-analysis: *MTHFR C677T* genotypes and risks of hypertensive disorders in pregnancy (HDP) (71 studies).

Genotype (Number of Studies)	HDP *N* = 8064 *n* (%)	Control *N* = 13,232 *n* (%)	Test of Association		
			Model Tested	Risk Ratio (95% CI)	*p*
*TT* (71)	1087 (13.48)	1410 (10.66)	Random	1.28 (1.15–1.43)	<0.0001
Caucasian (27)	425 (11.65)	700 (9.81)	Fixed	1.14 (1.00–1.30)	0.0474
Hispanic (7)	215 (28.10)	325 (29.15)	Fixed	0.97 (0.84–1.12)	0.6566
South American (4)	62 (16.40)	66 (11.89)	Fixed	1.40 (1.01–1.93)	0.0405
East Asian (17)	296 (23.59)	240 (11.82)	Fixed	1.75 (1.50–2.05)	<0.0001
South Asian (4)	15 (2.67)	31 (3.13)	Fixed	0.94 (0.49–1.81)	0.8606
Middle East (7)	54 (7.26)	46 (7.32)	Fixed	0.99 (0.67–1.45)	0.9482
African (5)	20 (2.81)	2 (0.26)	Fixed	5.82 (2.06–16.5)	0.0009
*CT* (71)	3142 (38.96)	5166 (39.04)	Random	1.01 (0.96–1.06)	0.7256
Caucasian (27)	1564 (42.87)	3001 (42.04)	Fixed	1.04 (0.98–1.10)	0.1913
Hispanic (7)	360 (47.06)	524 (47.00)	Fixed	1.00 (0.91–1.11)	0.9383
South American (4)	173 (45.77)	258 (46.49)	Fixed	0.94 (0.81–1.08)	0.3468
East Asian (17)	548 (43.67)	828 (40.79)	Random	1.00 (0.86–1.17)	0.9846
South Asian (4)	94 (16.76)	199 (20.08)	Fixed	0.77 (0.61–0.98)	0.0335
Middle East (7)	267 (35.89)	206 (32.80)	Fixed	1.07 (0.92–1.23)	0.3742
African (5)	136 (19.07)	150 (19.35)	Fixed	1.08 (0.88–1.32)	0.4717
*CC* (71)	3835 (47.56)	6656 (50.30)	Random	0.94 (0.90–0.98)	0.0017
Caucasian (27)	1659 (45.48)	3437 (48.15)	Fixed	0.94 (0.89–0.99)	0.0121
Hispanic (7)	190 (24.84)	266 (23.86)	Fixed	1.03 (0.88–1.21)	0.7027
South American (4)	143 (37.83)	231 (41.62)	Fixed	0.96 (0.81–1.13)	0.6263
East Asian (17)	411 (32.75)	962 (47.39)	Random	0.76 (0.67–0.87)	<0.0001
South Asian (4)	452 (80.57)	761 (76.79)	Random	1.06 (0.95–1.17)	0.3296
Middle East (7)	423 (56.85)	376 (59.87)	Fixed	0.96 (0.88–1.05)	0.4077
African (5)	557 (78.12)	623 (80.39)	Fixed	0.95 (0.91–1.00)	0.0441
*TT+CT* (71)	4229 (52.44)	6576 (49.70)	Random	1.07 (1.03–1.11)	0.0002
Caucasian (27)	1989 (54.52)	3701 (51.85)	Fixed	1.06 (1.01–1.11)	0.0116
Hispanic (7)	575 (75.16)	849 (76.14)	Fixed	0.99 (0.94–1.04)	0.6557
South American (4)	235 (62.17)	324 (58.38)	Fixed	1.03 (0.93–1.14)	0.6174
East Asian (17)	844 (67.25)	1068 (52.61)	Random	1.17 (1.08–1.27)	0.0002
South Asian (4)	109 (19.43)	230 (23.21)	Random	0.83 (0.56–1.22)	0.3382
Middle East (7)	321 (43.15)	252 (40.13)	Fixed	1.05 (0.93–1.19)	0.4069
African (5)	156 (21.88)	152 (19.61)	Fixed	1.21 (1.01–1.46)	0.0418
*CC+CT* (71)	6977 (86.52)	11,822(89.34)	Random	0.98 (0.96–0.99)	0.0023
Caucasian (27)	3223 (88.35)	6438 (90.19)	Fixed	0.98 (0.97–1.00)	0.0547
Hispanic (7)	550 (71.90)	790 (70.85)	Fixed	1.01 (0.96–1.07)	0.6426
South American (4)	316 (83.60)	489 (88.11)	Fixed	0.95 (0.90–0.99)	0.0475
East Asian (17)	959 (76.41)	1790 (88.18)	Random	0.89 (0.85–0.94)	<0.0001
South Asian (4)	546 (97.33)	960 (96.87)	Fixed	1.00 (0.98–1.02)	0.8593
Middle East (7)	690 (92.74)	582 (92.68)	Fixed	1.00 (0.97–1.03)	0.9471
African (5)	693 (97.19)	773 (99.74)	Random	0.98 (0.96–0.99)	0.0013
Subgroups					
*TT* risk > 1	4575 (56.74)	8472 (64.03)			
*TT+CT* (49)	2527 (55.23)	4289 (50.63)	Random	1.10 (1.05–1.15)	<0.0001
*CC+CT* (49)	3893 (80.09)	7675 (90.59)	Random	0.95 (0.93–0.97)	<0.0001
*TT* risk < 1	784 (9.72)	2529 (19.11)			
*TT+CT* (8)	309 (39.41)	1115 (44.09)	Fixed	0.90 (0.80–1.01)	0.0615
*CC+CT* (8)	728 (92.86)	2293 (90.67)	Fixed	1.03 (1.00–1.06)	0.0415
*TT* risk vary	2705 (33.54)	2231 (16.86)			
*TT+CT* (14)	1393 (51.50)	1172 (52.53)	Fixed	1.03 (0.98–1.09)	
*CC+CT* (14)	2356 (87.10)	1854 (83.10)	Fixed	1.01 (0.98–1.03)	0.211
					0.6066

Note: *TT* risk > 1, 21 countries: Finland, Denmark, Ireland, Netherlands, Slovakia, Hungary, Czech Republic, Italy, Spain, USA, Brazil, Ecuador, Peru, Japan, China, Indonesia, Sri Lanka, Turkey, Egypt, Zimbabwe, Tunisia; *TT* risk < 1, 5 countries: Australia, Germany, Austria, Croatia, India; *TT* risk varied around 1, 4 countries: UK, Mexico, Iran, South Africa.

**Table 3:** Meta-prediction: Death from air pollution (AP Death) on *MTHFR C677T* genotypes for controls (ct), hypertensive disorders in pregnancy (HDP), and HDP risks.

		Partition Tree							Tukey Test		
Variable	AICc	AP Death	Count	Mean	SD	Levels Compared	Difference	SE Difference	Lower CI	Upper CI	*p*
*TT+CT* % ct	610.933	2 and 3	42	48.445	19.033	4/3	6.064	5.335	−6.718	18.846	0.495
		4	29	54.167	14.505	4/2	5.490	4.766	−5.931	16.910	0.486
						2/3	0.547	5.490	−12.580	13.729	0.994
*TT+CT*% HDP	614.225	2 and 3	42	50.942	19.961	4/2	11.955	4.872	0.281	23.629	0.044
		4	29	61.951	13.875	4/3	9.618	5.453	−3.449	22.684	0.190
						3/2	2.338	5.612	−11.110	15.785	0.909
*CC*% ct	610.933	2 and 3	42	51.555	19.033	3/4	6.064	5.335	−6.718	18.846	0.495
		4	29	45.833	14.505	2/4	5.490	4.766	−5.931	16.910	0.486
						3/2	0.574	5.490	−12.580	13.729	0.994
CC% HDP	616.292	2 and 3	42	49.058	19.961	2/4	11.955	4.872	0.281	23.629	0.044
		4	29	38.049	13.875	3/4	9.618	5.453	−3.449	22.684	0.190
						2/3	2.338	5.612	−11.110	15.785	0.909
*CT*% ct	569.758	2 and 3	42	36.369	13.835	4/3	6.444	3.989	−3.114	16.002	0.246
		4	29	42.024	11.599	4/2	5.118	3.564	−3.421	13.658	0.328
						2/3	1.326	4.105	−8.511	11.162	0.944
*CT*% HDP	557.778	2 and 3	42	36.335	13.353	4/2	7.937	3.223	0.213	15.660	0.043
		4	29	43.339	8.922	4/3	5.634	3.608	−3.010	14.279	0.269
						3/2	2.302	3.713	−6.594	11.198	0.810
*TT*% ct	517.829	2	25	11.771	10.535	3/2	0.753	2.850	−6.074	7.581	0.962
		3 and 4	46	12.285	8.062	3/4	0.380	2.769	−6.254	7.014	0.990
						4/2	0.373	2.474	−5.554	6.301	0.988
*TT*% HDP	553.728	2 and 3	42	14.607	12.459	4/2	4.019	3.186	−3.615	11.653	0.422
		4	29	18.611	10.183	4/3	3.982	3.566	−4.562	12.527	0.507
						3/2	0.037	3.670	−8.756	8.831	0.999
RR *TT+CT*	4.424	2 and 3	42	1.075	0.228	4/2	0.131	0.066	−0.028	0.289	0.128
		4	29	1.184	0.261	4/3	0.078	0.074	−0.100	0.256	0.546
						3/2	0.052	0.076	−0.131	0.235	0.774
RR *CC*	−28.007	2	25	0.988	0.177	2/4	0.152	0.052	0.026	0.277	0.014
		3 and 4	46	0.864	0.201	2/3	0.079	0.060	−0.066	0.224	0.395
						3/4	0.073	0.059	−0.068	0.213	0.436
RR CT	49.064	2 and 3	42	1.006	0.277	4/2	0.113	0.090	−0.102	0.328	0.423
		4	29	1.106	0.388	4/3	0.081	0.100	−0.160	0.322	0.701
						3/2	0.032	0.103	−0.216	0.280	0.948
RR *TT*	143.232	2 and 3	36	1.193	0.661	4/3	0.547	0.229	−0.003	1.097	0.051
		4	29	1.587	0.755	4/2	0.296	0.199	−0.182	0.774	0.304
						2/3	0.251	0.241	−0.327	0.829	0.552

Note: 1. RR: Risk Ratio; 2. AP death: Death rates from air pollution, Levels per million: 2: ≤100, 3: 101–250, 4: >251; 3. AICc: Akaike’s information criterion correction.

## Supplementary Materials

The following are available online at http://www.mdpi.com/1660-4601/15/2/326/s1, Figure S1a. The percentage of *MTHFR 677 CT* and *TT* polymorphisms per control and hypertensive disorders in pregnancy case groups. Figure S1b. The percentage of *MTHFR 1298 AC* and *CC* polymorphisms per control and hypertensive disorders in pregnancy case groups. Figure S2a. Geographic information maps for % *MTHFR 677 TT* plus *CT* polymorphism per control and hypertensive disorders in pregnancy (HDP) groups, and HDP risks. Figure S2b. Geographic information maps for % *MTHFR 1298 CC* plus *AC* polymorphism per control and hypertension diseases in pregnancy (HDP) case groups, and HDP risks. Figure S3a. Forest plot for *MTHFR C677T* with types of hypertensive disorders in pregnancy for countries of TT risk > 1. Figure S3b. Forest plot for *MTHFR C677T* with types of hypertensive disorders in pregnancy for countries of TT risk < 1. Figure S3c. Forest plot for *MTHFR C677T* with types of hypertensive disorders in pregnancy for countries of TT risk ~ 1. Figure S4a. Forest plot for *MTHFR A1298C* with types of hypertensive disorders in pregnancy for countries of CC risk > 1. Figure S4b. Forest plot for *MTHFR A1298C* with types of hypertensive disorders in pregnancy for countries of CC risk < 1. Figure S5a. Heat maps of *MTHFR 677* homozygous TT genotypes for control and case groups in association with the rate of deaths from air pollution. Figure S5b. Heat maps of *MTHFR 677 TT* plus *CT* genotypes for control and case groups in association with the rate of deaths from air pollution. Table S1. Characteristics of studies for *MTHFR 677* and *1298* loci distributions (70 papers). Table S2a. Differences on risk ratio and odds ratio for *MTHFR C677T* polymorphism in hypertensive disorders in pregnancy (71 studies). Table S2b. Differences on risk ratio and odds ratio for *MTHFR A1298C* polymorphism in hypertensive disorders in pregnancy (11 studies). Table S3. Pooled meta-analysis: *MTHFR A1298C* polymorphism and risk of hypertensive disorders in pregnancy (11 studies). Table S4a. Pooled meta-analysis: *MTHFR C677T* polymorphism and risks of hypertensive disorders in pregnancy (HDP) per HDP types (71 studies). Table S4b. Pooled meta-analysis: *MTHFR A1298C* polymorphism and risk of hypertensive disorders in pregnancy (HDP) per HDP types (11 studies).
