# Supplementary material for: Meta-Prediction of MTHFR Gene Polymorphisms and Air Pollution on the Risk of Hypertensive Disorders in Pregnancy Worldwide
Source: Int J Environ Res Public Health. 2018 Feb 13;15(2):326. doi: 10.3390/ijerph15020326 (PMC5858395; doi:10.3390/ijerph15020326)
Supplement: Supplementary file 1 [file ijerph-15-00326-s001.pdf]

Table S1. Characteristics of studies for *MTHFR 677* and *1298* loci distributions (70 papers).

First Author (Reference number)	Year	Ethnicity-Country	MTHFR 677: Cases, Type, n (%)				MTHFR 677: Controls, source (s), n (%)					MTHFR 1298: Cases, n (%)			MTHFR 1298: Controls, n (%)				Quality Score
			Type	CC	CT	TT	S	CC	CT	TT	HWE	AA	AC	CC	AA	AC	CC	HWE	
**Australia**																			
Kaiser (16)	2001	Caucasian-Australia	1	71 (45.5)	66 (42.3)	19 (12.2)	1	37 (46.8)	31 (39.2)	11 (13.9)	Yes	NA							18 (5, 9, 4)
Kaiser (17)	2000	Caucasian-Australia	1	65 (44.2)	68 (46.3)	14 (9.5)	1	46 (42.2)	49 (45.0)	14 (12.8)	Yes	53 (36.0)	81 (55.2)	13 (8.8)	44 (40.4)	53 (48.6)	12 (11.0)	No	20 (5, 9, 6)
**Europe**																			
Hiltunen (18)	2009	Caucasian-Finland	1	150 (42.7)	84 (45.7)	14 (11.6)	1	380 (56.3)	263 (39.0)	32 (04.7)	Yes	NA							26 (8, 12, 6)
Jääskeläinen (19)	2006	Caucasian-Finland	1	78 (55.1)	43 (36.2)	12 (8.7)	1	64 (70.1)	42 (25.4)	6 (4.5)	Yes	NA							23 (7, 10, 6)
Laivuori (20)	2000	Caucasian-Finland	1	64 (46.3)	45 (43.3)	4 (10.4)	1	56 (47.3)	40 (43.3)	7 (9.4)	Yes	NA							23 (6, 10, 7)
Mislanova (21)	2011	Caucasian-Slovak	1	12 (42.8)	11 (39.3)	5 (17.8)	1	21 (52.5)	17 (42.5)	2 (5.0)	Yes	NA							22 (6, 10, 6)
Nagy (22)	2007	Caucasian-Hungary	1, 4<br>1<br>4	71 (43.3)<br>49 (48.5)<br>22 (34.9)	68 (41.5)<br>43 (42.5)<br>25 (39.6)	25 (15.2)<br>9 (8.9)<br>16 (25.4)	1	32 (43.8)<br>NA<br>NA	35 (47.9)	6 (8.2)	Yes	NA							24 (7, 11, 6)
Rigó, Jr. (23)	2000	Caucasian-Hungary	1	46 (38.3)	66 (55)	8 (6.7)	1	42 (41.6)	53 (52.5)	6 (5.9)	No	NA							18 (6, 7, 5)
Procházka (24)	2003	Caucasian-Czech	1	24 (63.1)	12 (31.6)	2 (5.3)	1	40 (80)	10 (20)	0 (0)	Yes	NA							21 (5, 10, 6)
Stonek (25)	2007	Caucasian-Austria	1	9 (36.0)	14 (56.0)	2 (8.0)	1	669 (48.0)	573 (41.0)	155 (11.0)	Yes	NA							24 (7, 10, 7)
Grandone (26)	1997	Caucasian-Italy	1	25 (26.6)	41 (43.6)	28 (29.8)	1	41 (31.8)	64 (49.6)	24 (18.6)	Yes	NA							22 (6, 10, 6)
Muetze (27)	2008	Caucasian-Germany	4	30 (42.3)	34 (47.9)	7 (9.9)	1	35 (44.3)	29 (36.7)	15 (19.0)	Yes	NA							21 (6, 9, 6)

First Author (Reference number)	Year	Ethnicity-Country	MTHFR 677									MTHFR 1298							Quality Score
			Cases, Type, n (%)				Controls, source (s), n (%)					Cases, n (%)			Controls, n (%)				
			Type	CC	CT	TT	S	CC	CT	TT	HWE	AA	AC	CC	AA	AC	CC	HWE	
**Europe**																			
Prasmusinto (28)	2002	Caucasian - Germany	1	7 (46.7)	7 (46.7)	1 (6.7)	1	12 (35.3)	15 (44.1)	7 (20.6)	Yes	NA							24 (6, 11, 7)
		Caucasian - Croatia	1	11 (44.0)	12 (48.0)	2 (7.6)	1	18 (47.3)	15 (39.5)	5 (13.2)	Yes								
		South Asia - Indonesia	1	34 (83.0)	6 (14.6)	1 (2.4)	1	22 (81.5)	5 (18.5)	0 (0.0)	Yes								
Lykke (29)	2012	Caucasian - Denmark	1, 4	113 (43.1)	118 (45.0)	31 (11.8)	1	906 (49.2)	793 (43.1)	143 (7.8)	Yes	NA							24 (7, 10, 7)
Demaat (30)	2004	Caucasian - Netherland	1	78 (46.8)	59 (37.6)	20 (12.7)	1	63 (40.1)	75 (47.8)	19 (12.1)	Yes	NA							22 (5, 11, 6)
Lachmeijer (31)	2001	Caucasian - Netherland	1, 4	22 (37.9)	21 (44.7)	4 (8.5)	1	58 (48.3)	51 (42.5)	11 (9.2)	Yes	18 (38.3)	22 (46.8)	7 (14.9)	45 (37.5)	64 (53.3)	11 (9.2)	Yes	20 (6, 8, 6)
Zusterzeel (14)	2000	Caucasian - Netherland	1, 4	72 (43.0)	74 (44.0)	21 (13.0)	1	205 (51.0)	162 (40.0)	36 (9.0)	Yes	91 (52.0)	68 (39.0)	17 (10.0)	179 (44.0)	186 (46.0)	38 (9.0)	Yes	23 (7, 10, 6)
			1	32 (42.0)	33 (43.0)	11 (14.0)	NA					45 (56.0)	26 (32.0)	10 (12.0)	NA				
			4	40 (44.0)	41 (45.0)	10 (11.0)	NA					46 (48.0)	42 (44.0)	7 (7.0)	NA				
Morrison (15)	2002	Caucasian - Scotland	3	284 (40.2)	347 (49.1)	76 (10.7)	1	81 (49.4)	66 (40.2)	17 (10.4)	Yes	NA							20 (5, 9, 6)
			1	169 (41.8)	193 (47.7)	42 (10.4)	NA												
			2	115 (37.9)	154 (50.8)	34 (11.2)	NA												
O'Shaughnessy (32)	1999	Caucasian-UK	1	138 (48.8)	114 (40.3)	31 (11.0)	1	51 (51.0)	37 (37.0)	12 (12.0)	Yes	NA							20 (7, 7, 6)
Murphy (33)	2000	Caucasian - Ireland	1	5 (45.4)	3 (27.3)	3 (27.3)	1	214 (39.6)	270 (50.0)	56 (10.4)	No	NA							20 (3,11, 6)
Pertegal (34)	2016	Caucasian - Spain	1	16 (30.2)	24 (45.3)	13 (24.5)	1	31 (43.1)	29 (40.3)	12 (16.7)	Yes	NA							23 (6, 10, 7)
Also-Rallo (35)	2005	Caucasian-Spain	1	11 (25.6)	24 (55.8)	8 (18.6)	1	38 (30.8)	57 (46.7)	27 (22.5)	Yes	24 (54.6)	19 (43.6)	0 (0.0)	70 (57.3)	41 (33.6)	11 (9.1)	Yes	24 (7, 11, 6)

First Author (Reference number)	Year	Ethnicity-Country	MTHFR 677									MTHFR 1298							Quality Score
			Cases, Type, n (%)				Controls, source (s), n (%)					Cases, n (%)			Controls, n (%)				
			Type	CC	CT	TT	S	CC	CT	TT	HWE	AA	AC	CC	AA	AC	CC	HWE	
**North America**																			
Powers (36)	2003	Caucasian - USA	1	14 (51.9)	0 (0.0)	13 (48.1)	1	15 (50.0)	0 (0.0)	15 (50.0)	No	NA							19 (5, 8, 6)
Kim (37)	2001	Caucasian - USA	1, 4	131 (46.6)	117 (41.6)	33 (11.7)	2	167 (46.4)	152 (42.2)	41 (11.4)	Yes	NA							25 (7, 11, 7)
			4	10 (47.6)	9 (42.9)	2 (9.5)			NA										
Livingstone (38)	2001	Caucasian - USA	1	66 (60.0)	34 (31.0)	10 (9.0)	1	61 (64.2)	27 (28.4)	7 (7.4)	Yes	NA							23 (7, 9, 7)
Powers (39)	1999	Caucasian - USA	3	46 (37.4)	58 (47.2)	19 (15.4)	1	54 (47.4)	46 (40.4)	14 (12.2)	Yes	NA							22 (7, 9, 6)
			1	35 (35.5)	49 (49.5)	15 (15.0)			NA										
			2	11 (47.0)	9 (38.3)	4 (14.7)			NA										
Hispanic																			
Vazquez-Alaniz (40)	2014	Mestizo - Mexican	3	59 (31.4)	91 (48.4)	38 (20.2)	1	54 (28.0)	97 (50.0)	43 (22.0)	Yes	NA							23 (7,10, 6)
			1	37 (32.7)	57 (50.5)	19 (16.8)			NA										
			2	22 (29.3)	34 (45.4)	19 (25.3)			NA										
Coral-Vázquez (41)	2013	Mestizo – Mexican	1	38 (16.5)	109 (47.4)	83 (36.1)	1	71 (20.2)	166 (47.2)	115 (32.7)	Yes	NA							22 (6, 9, 7)
Rojas (42)	2010	Mestizo – Mexican	1	8 (28.5)	9 (32.1)	11 (39.2)	1	12 (29.2)	19 (46.3)	10 (24.3)	Yes	NA							20 (4, 10, 6)
Canto (43)	2008	Mestizo – Mexican	1	36 (28.8)	66 (52.8)	23 (18.4)	1	61 (22.2)	131 (47.8)	82 (29.9)	Yes	NA							23 (6, 10, 7)
Dávalos (44)	2005	Mestizo – Mexican	1	13 (39.4)	14 (42.4)	6 (18.2)	1	24 (38.7)	27 (43.5)	11 (17.7)	Yes	NA							21 (7, 9, 5)
Pérez-Mutul (45)	2004	Mestizo - Mexican	1	33 (22.0)	66 (45.0)	49 (33.0)	1	36 (20.0)	80 (45.0)	61 (35.0)	Yes	NA							20 (6, 8, 6)
Perales Dávila (46)	2001	Mestizo-Mexican	1	3 (23.1)	5 (38.5)	5 (38.5)	1	8 (53.3)	4 (26.6)	3 (20.0)	Yes	NA							20 (4, 10, 6)

First Author (Reference number)	Year	Ethnicity-Country	MTHFR 677									MTHFR 1298							Quality Score
			Cases, Type, n (%)				Controls, source (s), n (%)					Cases, n (%)			Controls, n (%)				
			Type	CC	CT	TT	S	CC	CT	TT	HWE	AA	AC	CC	AA	AC	CC	HWE	
South American																			
Dusse (47)	2007	Mixed-Brazil	1	16 (53.3)	12 (40.0)	2 (6.7)	1	46 (53.5)	31 (37.3)	6 (7.2)	Yes	NA							20 (6, 10, 4)
Dalmáz (48)	2006	Mixed-Brazil	1	31 (41.3)	27 (36.0)	17 (22.7)	1	76 (52.4)	51 (35.2)	18 (12.4)	Yes	NA							20 (6, 8, 6)
Chedraui (49)	2014	Mixed - Ecuador	1	59 (39.3)	73 (48.7)	18 (12.0)	1	47 (31.3)	91 (60.7)	12 (8.0)	No	100 (66.7)	27 (18.0)	23 (15.3)	110 (73.3)	39 (26.0)	1 (0.7)	Yes	20 (6, 9, 5)
Williams (50)	2004	Mixed-Peru	1	37 (29.6)	61 (48.8)	25 (20.0)	1	62 (34.6)	85 (47.5)	30 (16.8)	Yes	NA							24 (6, 11, 7)
East Asia																			
Yoshida (51)	2008	East Asian-Japan	1	17 (32.7)	17 (32.7)	18 (34.6)	1	48 (42.5)	54 (47.8)	11 (9.7)	Yes	NA							20 (5, 9, 6)
Watanabe (52)	2001	East Asian-Japan	1	40 (30.0)	59 (44.0)	34 (26.0)	1	89 (40.0)	103 (46.0)	32 (14.0)	Yes	NA							15* (5, 6, 4)
Kobashi (53)	2000	East Asian-Japan	3	37 (36.6)	52 (51.5)	12 (11.9)	1	83 (38.6)	99 (46.0)	33 (15.3)	Yes	NA							21 (6, 9, 6)
			1	25 (34.2)	40 (54.8)	8 (11.0)	NA												
			2	12 (42.9)	12 (42.9)	4 (14.2)	NA												
Sohda (54)	1997	East Asian-Japan	1	19 (28.0)	32 (48.0)	16 (24.0)	1	38 (39.0)	49 (50.0)	11 (11.0)	Yes	NA							16* (5, 7, 4)
Su (55)	2011	East Asian-China	1	32 (59.2)	11 (20.4)	11 (20.4)	1	331 (64.5)	154 (30.0)	28 (5.5)	Yes	NA							22 (6, 10, 6)
Zhong (56)	2010	East Asian-China	3	33 (36.0)	44 (46.9)	16 (17.0)	1	26 (64.7)	8 (20.6)	6 (14.7)	No	NA							22 (7, 10, 5)
Shen (57)	2009	East Asian-China	3	20 (25.0)	42 (52.5)	18 (22.5)	1	30 (50.0)	21 (35.0)	9 (15.0)	Yes	NA							21 (6, 10, 5)
			1	12 (19.7)	35 (57.4)	14 (23.0)	NA												
			2	8 (42.1)	7 (36.8)	4 (21.1)	NA												

First Author (Reference number)	Year	Ethnicity-Country	MTHFR 677									MTHFR 1298							Quality Score
			Cases, Type , n (%)				Controls, source (s), n (%)					Cases, n (%)			Controls, n (%)				
			Type	CC	CT	TT	S	CC	CT	TT	HWE	AA	AC	CC	AA	AC	CC	HWE	
**East Asia**																			
Ding (58)	2008	East Asian-China	2	20 (21.8)	37 (40.2)	35 (38.0)	1	18 (20.2)	55 (61.8)	16 (18.0)	No	NA							19 (4, 9, 6)
Wang (59)	2008	East Asian-China	1	6 (14.3)	19 (45.2)	17 (40.5)	1	13 (20.3)	40 (62.5)	11 (17.2)	No	NA							21 (5, 11, 5)
Zhang (60)	2007	East Asian-China	1	12 (22.7)	21 (39.6)	20 (37.7)	1	10 (20.4)	30 (61.2)	9 (18.4)	Yes	NA							22 (6, 10, 6)
Wang (61)	2006	East Asian-China	3	26 (48.1)	22 (40.7)	6 (11.1)	1	89 (71.2)	27 (21.6)	9 (7.2)	No	NA							19 (3, 10, 6)
Tian (62)	2005	East Asian-China	3	34 (55.7)	20 (32.7)	7 (11.5)	1	40 (71.5)	12 (21.4)	4 (7.1)	No	NA							23 (7, 11, 5)
			1	22 (56.9)	12 (30.8)	5 (12.8)			NA										
			2	12 (54.5)	8 (36.4)	2 (9.1)			NA										
Niu (63)	2004	East Asian-China	1, 4	6 (8.2)	45 (61.6)	22 (30.2)	1	12 (16.2)	43 (58.1)	19 (25.7)	Yes	NA							20 (5, 10, 5)
Wang (64)	2004	East Asian-China	3	53 (53.5)	31 (31.3)	15 (15.2)	1	25 (46.3)	24 (44.4)	5 (9.3)	Yes	NA							20 (5, 9, 6)
Fu (65)	2003	East Asian-China	2	24 (23.5)	53 (52.0)	25 (24.5)	1	46 (46.0)	40 (40.0)	14 (14.0)	Yes	NA							18 (4, 10, 4)
Wei (66)	2001	East Asian-China	2	23 (54.7)	13 (31.0)	6 (14.3)	1	20 (55.6)	11 (30.5)	5 (13.9)	Yes	NA							22 (6, 11, 5)
Li (67)	2000	East Asian-China	1	9 (16.0)	30 (53.0)	18 (31.0)	2	44 (37.0)	58 (49.0)	18 (15.0)	Yes	NA							17 (4, 8, 5)
Liao (68)	2005	East Asian-China	2		NA		1		NA			31 (57.4)	20 (37.0)	3 (5.6)	60 (60.0)	36 (36.0)	4 (4.0)	Yes	22 (7, 10, 5)
**South Asia**																			
Kaur (69)	2013	South Asian-India	1	122 (84.1)	19 (13.1)	4 (2.8)	1	463 (78.1)	109 (18.4)	21 (3.5)	No	NA							20 (5, 9, 6)
Aggarwal (70)	2011	South Asian-India	1	160 (80.0)	33 (16.5)	7 (3.5)	1	134 (67.0)	58 (29.0)	8 (4.0)	Yes	NA							24 (7, 10, 7)
Dissanayake (71)	2012	South Asian-Sri Lanka	1	136 (77.7)	36 (20.6)	3 (1.7)	1	142 (83.0)	27 (15.8)	2 (1.2)	Yes	71 (41.0)	89 (51.4)	13 (7.5)	76 (44.4)	83 (48.5)	12 (7.0)	Yes	22 (6, 10, 6)

First Author (Reference number)	Year	Ethnicity-Country	MTHFR 677									MTHFR 1298							Quality Score
			Cases, type, n (%)				Controls, source (s), n (%)					Cases, n (%)			Controls, n (%)				
			Type	CC	CT	TT	S	CC	CT	TT	HWE	AA	AC	CC	AA	AC	CC	HWE	
**Middle Eastern**																			
Salimi (72)	2015	Mid-Eastern-Iran	1	124 (64.6)	60 (31.2)	8 (4.2)	1	136 (69.4)	51 (26.0)	9 (4.6)	Yes	124 (64.6)	58 (30.2)	10 (5.2)	146 (74.5)	43 (22.0)	7 (3.5)	Yes	24 (7, 11, 6)
Rahimi (73)	2013	Mid-Eastern-Iran	1	110 (55.6)	72 (36.4)	16 (8.1)	1	52 (51.5)	45 (44.6)	4 (4.0)	Yes	NA							20 (7, 7, 6)
Saravani (74)	2011	Mid-Eastern-Iran	1	82 (77.0)	23 (22.0)	1 (1.0)	1	78 (73.0)	21 (19.5)	8 (7.5)	No	NA							
Deveer (75)	2013	Mid-Eastern-Turkey	1	29 (58.0)	16 (32.0)	5 (10.0)	1	26 (52.0)	18 (36.0)	6 (12.0)	Yes	28 (36.0)	25 (50.0)	7 (14.0)	20 (40.0)	26 (52.0)	4 (8.0)	Yes	19 (5, 9, 5)
Dogan (76)	2011	Mid-Eastern-Turkey	4	16 (47.1)	15 (44.1)	3 (8.8)	1	17 (68.0)	7 (28.0)	1 (4.0)	Yes	NA							22 (6, 10, 6)
Demir (77)	2006	Mid-Eastern-Turkey	2	31 (34.4)	49 (54.5)	10 (11.1)	1	43 (42.2)	47 (46.1)	12 (11.8)	Yes	NA							20 (6, 8, 6)
Yilmaz (78)	2004	Caucasian-Turkey	1	29 (45.3)	28 (43.8)	7 (10.9)	1	24 (51.1)	17 (36.2)	6 (12.7)	Yes	NA							20 (6, 8, 6)
Africa																			
Ibrahim (79)	2012	Mid-Eastern-Egypt	1	9 (20.5)	20 (45.4)	15 (34.1)	1	16 (36.4)	28 (63.6)	0 (0.0)	No	NA							22 (5, 11, 6)
Klai (80)	2012	African-Tunisia	1	22 (40.9)	20 (54.6)	2 (4.6)	2	61 (61.0)	39 (39.0)	0 (0.0)	No	40 (90.9)	0 (0.0)	4 (9.1)	93 (93.0)	7 (7.0)	0 (0.0)	Yes	21 (5, 10, 6)
Rajkovic (81)	2000	African-Zimbabwe	1	142 (83.0)	28 (16.4)	1 (0.6)	1	151 (82.5)	32 (17.5)	0 (0.0)	Yes	NA							23 (7, 10, 6)
Pergoraro (82)	2004	African-South Africa	3	298 (85.4)	50 (14.3)	1 (0.3)	1	298 (88.0)	38 (11.0)	2 (1.0)	Yes	265 (75.9)	80 (22.9)	4 (0.2)	263 (78.0)	67 (20.0)	8 (2.0)	Yes	22 (7, 9, 6)
			1	232 (86.0)	38 (14.0)	1 (0.0)	NA					213 (79.0)	55 (20.0)	3 (1.0)	NA				
			2	66 (85.0)	12 (15.0)	0 (0.0)	NA					52 (67.0)	25 (32.0)	1 (1.0)	NA				
Chikosi (83)	1999	African-South Africa	1	86 (82.9)	18 (17.1)	1 (1.0)	1	97 (88.2)	13 (11.8)	0 (0.0)	Yes	NA							16 (6, 6, 4)

Type (Type of hypertensive disorders in pregnancy): 1 = preeclampsia, and eclampsia, 2 = gestational hypertension, 3 = mixed (include 1 & 2), and 4= hemolysis, elevated liver enzymes and low platelet count (HELLP) syndrome,

S (Sources of controls): 1 = healthy pregnancy, 2 = healthy female adults, 3= healthy general people

HWE (Hardy Weinberg Equilibrium) updated from the original report based on our calculations using the formula available at http://www.koonec.com/k-blog/2010/06/20/hardy-weinberg-equilibrium-calculator.

Quality score ranges: 0 - 28 (external validity, 0 – 9; Internal Validity, 0 – 12; report quality, 0 -7). NA: Not available. *: Brief Report.

Reference List of Studies included in the Meta-analysis

12 Meta-analysis Papers

1. Wang X-m, Wu H-y, Qiu X-j. Methylenetetrahydrofolate reductase (MTHFR) gene C677T polymorphism and risk of preeclampsia: an updated meta-analysis based on 51 studies. Arch Med Res 2013;44:159-68.
2. Xia X-p, Chang W-w, Cao Y-x. Meta-analysis of the methylenetetrahydrofolate reductase C677T polymorphism and susceptibility to pre-eclampsia. Hypertens Res 2012;35:1129-34.
3. Lin J, August P. Genetic thrombophilias and preeclampsia: a meta-analysis. Obstet Gynecol 2005;105:182-92.
4. Li X, Luo YL, Zhang QH, Mao C, Wang XW, Liu S et al. Methylenetetrahydrofolate reductase gene C677T, A1298C polymorphisms and pre-eclampsia risk: a meta-analysis. Mol Biol Rep 2014;41:5435-48.
5. Kosmas IP, Tatsioni A, Ioannidis JP. Association of C677T polymorphism in the methylenetetrahydrofolate reductase gene with hypertension in pregnancy and pre-eclampsia: a meta-analysis. J Hypertens 2004;22:1655–62.
6. Yang B, Fan S, Zhi X, Li Y, Liu Y, Wang D et al. Associations of MTHFR gene polymorphisms with hypertension and hypertension in pregnancy: a meta-analysis from 114 studies with 15411 cases and 21970 controls. PLoS One 2014;9:e87497.
7. Reilly R, McNulty H, Pentieva K, Strain JJ, Ward M. MTHFR 677TT genotype and disease risk: is there a modulating role for B-vitamins? Proc Nutr Soc 2014;73:47-56.
8. Niu W, You Y, Qi Y. Strong association of methylenetetrahydrofolate reductase gene C677T polymorphism with hypertension and hypertension-in-pregnancy in Chinese: a meta-analysis. J Hum Hypertens 2012;26:259-67.
9. Qian X, Lu Z, Tan M, Liu H, Lu D. A meta-analysis of association between C677T polymorphism in the methylenetetrahydrofolate reductase gene and hypertension. Eur J Hum Genet 2007;15:1239-45.
10. Wu X, Yang K, Tang X, Sa Y, Zhou R, Liu J et al. Folate metabolism gene polymorphisms MTHFR C677T and A1298C and risk for preeclampsia: a meta-analysis. J Assist Reprod Genet 2015;32:797-805.
11. Tempfer C. SC, Riener E.-K., Hefler L.A., Keck C. . Polymorphisms for risk assessment of pregnancy-associated disorders - Review of the literature and Meta-analysis. . Geburtshilfe

Frauenheilkd 2004;64:1080-9.

12. Refsum H, Nurk E, Smith AD, Ueland PM, Gjesdal CG, Bjelland I et al. The Hordaland Homocysteine Study: a community-based study of homocysteine, its determinants, and associations with disease. J Nutr 2006;136:1731s-40s.

1 Papers with Duplicate Use of Data on Gene Counts

13. Prasmusinto D, Skrablin S, Fimmers R, van der Ven K. Ethnic differences in the association of factor V Leiden mutation and the C677T methylenetetrahydrofolate reductase gene polymorphism with preeclampsia. Eur J Obstet Gynecol Reprod Biol 2004;112:162-9.

70 Papers included in Meta-analysis

2 Meta-analysis Papers had individual data

14. Zusterzeel PL, Visser W, Blom HJ, Peters WH, Heil SG, Steegers EA. Methylenetetrahydrofolate reductase polymorphisms in preeclampsia and the HELLP syndrome. Hypertens Pregnancy 2000;19:299-307.

15. Morrison E, Miedzybrodzka Z, Campbell D, Haites N, Wilson B, Watson M et al. Prothrombotic genotypes are not associated with pre-eclampsia and gestational hypertension: results from a large population-based study and systematic review. Thromb Haemost 2002;87:779-85.

68 case control Papers

16. Kaiser T, Brennecke SP, Moses EK. C677T methylenetetrahydrofolate reductase polymorphism is not a risk factor for pre-eclampsia/eclampsia among Australian women. Hum Hered 2001;51:20-2.

17. Kaiser T, Brennecke SP, Moses EK. Methylenetetrahydrofolate reductase polymorphisms are not a risk factor for pre-eclampsia/eclampsia in Australian women. Gynecol Obstet Invest 2000;50:100-2.

18. Hiltunen LM, Laivuori H, Rautanen A, Kaaja R, Kere J, Krusius T et al. Blood group AB and factor V Leiden as risk factors for pre-eclampsia: a population-based nested case-control study. Thromb Res 2009;124:167-73.

19. Jaaskelainen E, Keski-Nisula L, Toivonen S, Romppanen EL, Helisalmi S, Punnonen K et al. MTHFR C677T polymorphism is not associated with placental abruption or preeclampsia in Finnish women. Hypertens Pregnancy 2006;25:73-80.

20. Laivuori H, Kaaja R, Ylikorkala O, Hiltunen T, Kontula K. 677 C-->T polymorphism of the

methylenetetrahydrofolate reductase gene and preeclampsia. Obstet Gynecol 2000;96:277-80.

21. Mislanova C, Martsenyuk O, Huppertz B, Obolenskaya M. Placental markers of folate-related metabolism in preeclampsia. Reproduction 2011;142:467-76.

22. Nagy B, Hupuczi P, Papp Z. High frequency of methylenetetrahydrofolate reductase 677TT genotype in Hungarian HELLP syndrome patients determined by quantitative real-time PCR. J Hum Hypertens 2007;21:154-8.

23. Rigo J, Jr., Nagy B, Fintor L, Tanyi J, Beke A, Karadi I et al. Maternal and neonatal outcome of preeclamptic pregnancies: the potential roles of factor V Leiden mutation and 5,10 methylenetetrahydrofolate reductase. Hypertens Pregnancy 2000;19:163-72.

24. Prochazka M, Krcova V, Kudela M, Slavik L. Factor V. Leiden, Prothrombin and Methylenetetrahydrofolate Reductase Gen Mutations in Patients with Preeclampsia. Ceska Gynekol 2003;68:162-6.

25. Stonek F, Hafner E, Philipp K, Hefler LA, Bentz EK, Tempfer CB. Methylenetetrahydrofolate reductase C677T polymorphism and pregnancy complications. Obstet Gynecol 2007;110:363-8.

26. Grandone E, Margaglione M, Colaizzo D, Cappucci G, Paladini D, Martinelli P et al. Factor V Leiden, C > T MTHFR polymorphism and genetic susceptibility to preeclampsia. Thromb Haemost 1997;77:1052-4.

27. Muetze S, Leeners B, Ortlepp JR, Kuse S, Tag CG, Weiskirchen R et al. Maternal factor V Leiden mutation is associated with HELLP syndrome in Caucasian women. Acta Obstet Gynecol Scand 2008;87:635-42.

28. Prasmusinto D, Skrablin S, Hofstaetter C, Fimmers R, van der Ven K. The methylenetetrahydrofolate reductase 677 C-->T polymorphism and preeclampsia in two populations. Obstet Gynecol 2002;99:1085-92.

29. Lykke JA, Bare LA, Olsen J, Lagier R, Arellano AR, Tong C et al. Thrombophilias and adverse pregnancy outcomes: results from the Danish National Birth Cohort. J Thromb Haemost 2012;10:1320-5.

30. De Maat MP, Jansen MW, Hille ET, Vos HL, Bloemenkamp KW, Buitendijk S et al. Preeclampsia and its interaction with common variants in thrombophilia genes. J Thromb Haemost 2004;2:1588-93.

31. Lachmeijer AM, Arngrimsson R, Bastiaans EJ, Pals G, ten Kate LP, de Vries JI et al. Mutations in the gene for methylenetetrahydrofolate reductase, homocysteine levels, and vitamin status in women with a history of preeclampsia. Am J Obstet Gynecol 2001;184:394-402.

32. O'Shaughnessy KM, Fu B, Ferraro F, Lewis I, Downing S, Morris NH. Factor V Leiden and thermolabile methylenetetrahydrofolate reductase gene variants in an East Anglian preeclampsia cohort. Hypertension 1999;33:1338-41.

33. Murphy RP, Donoghue C, Nallen RJ, D'Mello M, Regan C, Whitehead AS et al. Prospective evaluation of the risk conferred by factor V Leiden and thermolabile methylenetetrahydrofolate reductase polymorphisms in pregnancy. Arterioscler Thromb Vasc Biol 2000;20:266-70.

34. Pertegal M, Fenoy FJ, Hernandez M, Mendiola J, Delgado JL, Bonacasa B et al. Fetal Val108/158Met catechol-O-methyltransferase (COMT) polymorphism and placental COMT activity are associated with the development of preeclampsia. Fertil Steril 2016;105:134-43.e1-3.

35. Also-Rallo E, Lopez-Quesada E, Urreizti R, Vilaseca MA, Lailla JM, Balcells S et al. Polymorphisms of genes involved in homocysteine metabolism in preeclampsia and in uncomplicated pregnancies. Eur J Obstet Gynecol Reprod Biol 2005;120:45-52.

36. Powers RW, Dunbar MS, Gallaher MJ, Roberts JM. The 677 C-T methylenetetrahydrofolate reductase mutation does not predict increased maternal homocysteine during pregnancy. Obstet Gynecol 2003;101:762-6.

37. Kim YJ, Williamson RA, Murray JC, Andrews J, Pietscher JJ, Peraud PJ et al. Genetic susceptibility to preeclampsia: roles of cytosineto-thymine substitution at nucleotide 677 of the gene for methylenetetrahydrofolate reductase, 68-base pair insertion at nucleotide 844 of the gene for cystathionine beta-synthase, and factor V Leiden mutation. Am J Obstet Gynecol 2001;184:1211-7.

38. Livingston JC, Barton JR, Park V, Haddad B, Phillips O, Sibai BM. Maternal and fetal inherited thrombophilias are not related to the development of severe preeclampsia. Am J Obstet Gynecol 2001;185:153-7.

39. Powers RW, Minich LA, Lykins DL, Ness RB, Crombleholme WR, Roberts JM. Methylenetetrahydrofolate reductase polymorphism, folate, and susceptibility to preeclampsia. J Soc Gynecol Investig 1999;6:74-9.

40. Vazquez-Alaniz F, Lumbreras-Marquez MI, Sandoval-Carrillo AA, Aguilar-Duran M, Mendez-Hernandez EM, Barraza-Salas M et al. Association of COMT G675A and MTHFR C677T polymorphisms with hypertensive disorders of pregnancy in Mexican mestizo population. Pregnancy Hypertens 2014;4:59-64.

41. Coral-Vazquez RM, Romero Arauz JF, Canizales-Quinteros S, Coronel A, Valencia Villalvazo EY, Hernandez Rivera J et al. Analysis of polymorphisms and haplotypes in genes associated with vascular

tone, hypertension and oxidative stress in Mexican-Mestizo women with severe preeclampsia. Clin Biochem 2013;46:627-32.

42. Rojas JC, Luna M, Rangel-Nava H, Banos D, Collados MT. Genetic thrombophilia and markers of endothelial activation in patients with preeclampsia. Ginecol Obstet Mex 2010;78:401-9.

43. Canto P, Canto-Cetina T, Juarez-Velazquez R, Rosas-Vargas H, Rangel-Villalobos H, Canizales-Quinteros S et al. Methylenetetrahydrofolate reductase C677T and glutathione S-transferase P1 A313G are associated with a reduced risk of preeclampsia in Maya-Mestizo women. Hypertens Res 2008;31:1015-9.

44. Davalos IP, Moran MC, Martinez-Abundis E, Gonzalez-Ortiz M, Flores-Martinez SE, Machorro V et al. Methylenetetrahydrofolate reductase C677T polymorphism and Factor V Leiden variant in Mexican women with preeclampsia/eclampsia. Blood Cells Mol Dis 2005;35:66-9.

45. Perez-Mutul J, Gonzalez-Herrera L, Sosa-Cabrera T, Martinez-Olivares R. A mutation in the 5,10-methylenetetrahydrofolate reductase gene is not associated with preeclampsia in women of southeast Mexico. Arch Med Res 2004;35:231-4.

46. Perales Davila J, Martinez de Villarreal LE, Triana Saldana H, Saldivar Rodriguez D, Barrera Saldana H, Rojas Martinez A et al. [Folic acid levels, homocysteine and polymorphism of methylenetetrahydrofolate reductase enzyme (MTHFR) in patients with pre-eclampsia and eclampsia]. Ginecol Obstet Mex 2001;69:6-11.

47. Dusse LM, Carvalho M, Braganca WF, Paiva SG, Godoi LC, Guimaraes DA et al. Inherited thrombophilias and pre-eclampsia in Brazilian women. Eur J Obstet Gynecol Reprod Biol 2007;134:20-3.

48. Dalmaz CA, Santos KG, Botton MR, Tedoldi CL, Roisenberg I. Relationship between polymorphisms in thrombophilic genes and preeclampsia in a Brazilian population. Blood Cells Mol Dis 2006;37:107-10.

49. Chedraui P, Salazar-Pousada D, Villao A, Escobar GS, Ramirez C, Hidalgo L et al. Polymorphisms of the methylenetetrahydrofolate reductase gene (C677T and A1298C) in nulliparous women complicated with preeclampsia. Gynecol Endocrinol 2014;30:392-6.

50. Williams MA, Sanchez SE, Zhang C, Bazul V. Methylenetetrahydrofolate reductase 677 C-->T polymorphism and plasma folate in relation to pre-eclampsia risk among Peruvian women. J Matern Fetal Neonatal Med 2004;15:337-44.

51. Yoshida A, Miura K, Nakayama D, Masuzaki H. Correlation between preeclampsia and prevalence of polymorphism of angiotensinogen, methyleneteterahydrofolate reductase and factor V, prothrombin

genes among Japanese women. Acta Med Nagasaki 2008;53:37-41.

52. Watanabe H, Hamada H, Yamakawa-Kobayashi K, Yoshikawa H, Arinami T. Evidence for an association of the R485K polymorphism in the coagulation factor V gene with severe preeclampsia from screening 35 polymorphisms in 27 candidate genes. Thromb Haemost 2001;86:1594-5.

53. Kobashi G, Yamada H, Asano T, Nagano S, Hata A, Kishi R et al. Absence of association between a common mutation in the methylenetetrahydrofolate reductase gene and preeclampsia in Japanese women. Am J Med Genet 2000;93:122-5.

54. Sohda S, Arinami T, Hamada H, Yamada N, Hamaguchi H, Kubo T. Methylenetetrahydrofolate reductase polymorphism and pre-eclampsia. J Med Genet 1997;34:525-6.

55. Nianjun S, Bing L, Jianhuai F. Study on the relationship between N5, 10-methylenetetrahydrofolate reductase gene and endothelial nitric oxide synthase gene polymorphisms and preeclampsia and eclampsia in the Han nationality women of Guangdong. Progress in Obstetrics and Gynecology 2011;20:199-202.

56. Zhong L, Feng Z, Zhu J, Wang R, Jiang J. Research on polymorphism of homocysteic acid (Hcy) metabolic enzymes genes among patients with pregnancy hypertension. Chin J Health Lab Tech 2010;20:2128-30.

57. Shen X, Huang Y, Tang S, Zhang C, Chen W. The relationship between the polymorphism of MTHFR gene and preeclampsia. J Pract Obstet Gynecol 2009;25:236-8 [in Chinese].

58. Ding YS, Guan LX, Wang YH. Study on MTHFR gene and eNOS gene polymorphisms in pregnancy-induced hypertension in the Hans of Weifang area. Chin J Birth Health Hered 2008;16:12-4 [in Chinese].

59. Wang SM, Wang LG, Liu XJ, Wu A, Yu J, Shi H. Investigation on the association between MTHFR gene C677T polymorphism and pre-eclampsia. Maternal and Child Health Care of China 2008;23:552-4 [in Chinese].

60. Zhang Z, Zhang R, Liu A, Xu Q, Liu Z. Study on eNOS gene and MTHFR gene polymorphisms in preeclampsia. Chin J Birth Health Hered 2007;15:21-4 [in Chinese].

61. Wang SM, Shen R, Shi XY, Jiang T, Liu XM, Shi YL et al. The role of homocysteine metabolism related enzymes gene polymorphisms on pregnancy-induced hypertension. Chin J Prep Genetic. 2006;14:15-7 [in Chinese].

62. Tian G, She D, Qi Q. Genetic research between gene polymorphism of homocysteine metabolism related enzymes and pre-eclampsia. Chin J Thromb Haemost. 2005;11:197-9 [in Chinese].

63. Niu XM, Zhang XY. The relationship between plasma homocysteine metabolism and gestational hypertension. Chin J Obstet Gynecol. 2004;39:347-8 [in Chinese].

64. Wang H, Li C, Wang Z, Yang F. Relationships between polymorphisms of angiotensin-converting enzyme and methylenetetrahydrofolate reductase genes and genetic susceptibility to pregnancy induced hypertension. Chin J Obstet Gynecol. 2004;39:369-72 [in Chinese].

65. Fu F, Liu H, Liao DL, Xiong LN, He XJ, Zhang W et al. Investigation of the relationship between polymorphism of methylenetetrahydrofolate reductase and pregnancy induced hypertension syndrome. Jiangxi Med J. 2003;38:401-3 [in Chinese].

66. Wei S, Zheng J, Shi D, Zou L, Bi L. Relationship between pregnancy induced hypertension and the polymorphisms of MTHFR gene and plasma homocysteine levels. Chin J Mod Med. 2001;11:10-9 [in Chinese].

67. Li K, Zheng D, Xue Y, Sun Y, Chen L, Guo J et al. The common C677T polymorphism in the methylenetetrahydrofolate reductase gene is associated with neural tube defects and preeclampsia. Chin J Med Genet. 2000;17:76-8 [in Chinese].

68. Liao Z, Tian G, Gao M, Chen S. The relationship between polymorphism of methylenetetrahydrofolate reductase (MTHFR1298) gene and pregnancy induced hypertension syndrome. Anat Res. 2005;27:94-6 [in Chinese].

69. Kaur L, Puri M, Kaushik S, Sachdeva MP, Trivedi SS, Saraswathy KN. Genetic thromobophilia in pregnancy: a case-control study among North Indian women. J Thromb Thrombolysis 2013;35:250-6.

70. Aggarwal S, Dimri N, Tandon I, Agarwal S. Preeclampsia in North Indian women: the contribution of genetic polymorphisms. J Obstet Gynaecol Res 2011;37:1335-41.

71. Dissanayake VH, Sirisena ND, Weerasekera LY, Gammulla CG, Seneviratne HR, Jayasekara RW. Candidate gene study of genetic thrombophilic polymorphisms in pre-eclampsia and recurrent pregnancy loss in Sinhalese women. J Obstet Gynaecol Res 2012;38:1168-76.

72. Salimi S, Saravani M, Yaghmaei M, Fazlali Z, Mokhtari M, Naghavi A et al. The early-onset preeclampsia is associated with MTHFR and FVL polymorphisms. Arch Gynecol Obstet 2015;291:1303-12.

73. Rahimi Z, Malek-Khosravi S, Rahimi Z, Jalilvand F, Parsian A. MTHFR C677T and eNOS G894T variants in preeclamptic women: Contribution to lipid peroxidation and oxidative stress. Clin Biochem 2013;46:143-7.

74. Saravani M, Salimi S, Yaghmaei M, Mokhtari M, Jafari M. Association between methylene

tetrahydrofolate reductase gene C677T polymorphism with preeclampsia in south-east of Iran. Zahedan Journal of Research in Medical Sciences 2011;13:39-43.

75. Deveer R, Engin-Ustun Y, Akbaba E, Halisdemir B, Cakar E, Danisman N et al. Association between pre-eclampsia and inherited thrombophilias. Fetal Pediatr Pathol 2013;32:213-7.

76. Dogan OO, Simsek Y, Celen S, Danisman N. Frequency of hereditary thrombophilia, anticoagulant activity, and homocysteine levels in patients with hemolysis, elevated liver functions and low thrombocyte count (HELLP) syndrome. J Obstet Gynaecol Res 2011;37:527-33.

77. Demir SC, Evruke C, Ozgunen T, Kadayifci O, Altintas U, Kokangul S. The relationship between pregnancy induced hypertension and congenital thrombophilia. Saudi Med J 2006;27:1161-6.

78. Yilmaz H, Unlucerci Y, Gurdol F, Isbilen E, Isbir T. Association of pre-eclampsia with hyperhomocysteinaemia and methylenetetrahydrofolate reductase gene C677T polymorphism in a Turkish population. Aust N Z J Obstet Gynaecol 2004;44:423-7.

79. Ibrahim ZM, Metawie MAE, El-Baz AM, El-Bahie MA. Methylenetetrahydrofolate C677T polymorphism and pre-eclamptic Egyptian women. Middle East Fertil Soc J 2012;17:105-10.

80. Klai S, Fekih-Mrissa N, El Housaini S, Kaabechi N, Nsiri B, Rachdi R et al. Association of MTHFR A1298C polymorphism (but not of MTHFR C677T) with elevated homocysteine levels and placental vasculopathies. Blood Coagul Fibrinolysis 2011;22:374-8.

81. Rajkovic A, Mahomed K, Rozen R, Malinow MR, King IB, Williams MA. Methylenetetrahydrofolate reductase 677 C --> T polymorphism, plasma folate, vitamin B(12) concentrations, and risk of preeclampsia among black African women from Zimbabwe. Mol Genet Metab 2000;69:33-9.

82. Pegoraro RJ, Chikosi A, Rom L, Roberts C, Moodley J. Methylenetetrahydrofolate reductase gene polymorphisms in black South Africans and the association with preeclampsia. Acta Obstet Gynecol Scand 2004;83:449-54.

83. Chikosi A, Moodley J, Pegoraro R, Lanning P, Rom L. 5, 10 methylenetetrahydrofolate reductase polymorphism in black South African women with pre-eclampsia. Br J Obstet Gynaecol 1999;106:1219-20.

Table S2a. Differences on risk ratio and odds ratio for *MTHFR C677T* polymorphism in hypertensive disorders in pregnancy (71 studies).

Genotype (number of studies)	Case N=8064 n (%)	Control N=13232 n (%)	Ratio Type	Test of Heterogeneity			Statistical Model	Test of Association	
				Q	p	I^2		Relative risk Ratio (95% CI)	p
TT (71)	1087 (13.48)	1410 (10.66)	Risk	107.30	.0028	34.8%	Random	1.28 (1.15 - 1.43)	<.0001
			Odds	108.06	.0024	35.2%	Random	1.34 (1.17 - 1.53)	<.0001
CT (71)	3142 (38.96)	5166 (39.04)	Risk	103.13	.0049	33.1%	Random	1.01 (0.96 - 1.06)	.7256
			Odds	106.52	.0025	35.2%	Random	1.02 (0.93 - 1.11)	.7059
CC (71)	3835 (47.56)	6656 (50.30)	Risk	120.18	.0002	41.8%	Random	0.94 (0.90 - 0.98)	.0017
			Odds	109.86	.0017	36.3%	Random	0.85 (0.78 - 0.93)	.0004
TT +CT (71)	4229 (52.44)	6576 (49.70)	Risk	106.43	.0033	34.2%	Random	1.07 (1.03 - 1.11)	.0002
			Odds	109.86	.0017	36.3%	Random	1.17 (1.07 - 1.28)	.0004
CC+CT (71)	6977 (86.52)	11822 (89.34)	Risk	185.55	<.0001	62.3%	Random	0.98 (0.96 - 0.99)	.0023
			Odds	108.06	.0024	35.2%	Random	0.75 (0.66 - 0.85)	<.0001

Table S2b. Differences on risk ratio and odds ratio for *MTHFR A1298C* polymorphism in hypertensive disorders in pregnancy (11 studies).

Genotype (number of studies)	Case N=1425 n (%)	Control N=1859 n (%)	Ratio Type	Test of Heterogeneity			Statistical Model	Test of Association	
				Q	p	I^2		Relative risk Ratio (95% CI)	p
CC (11)	101 (7.08)	108(5.81)	Risk	19.83	.0309	49.6%	Random	1.30 (0.82 - 2.07)	.2586
			Odds	20.62	.0239	51.5%	Random	1.35 (0.81 - 2.25)	.244
AC (11)	489 (34.27)	645 (34.70)	Risk	14.81	.1393	32.5%	Fixed	1.01 (0.92 - 1.11)	.8107
			Odds	15.04	.1307	33.5%	Fixed	1.02 (0.87 - 1.19)	.8384
AA (11)	835 (58.51)	1106 (59.49)	Risk	7.97	.6321	0%	Fixed	0.96 (0.91 - 1.02)	.1671
			Odds	8.64	.5666	0%	Fixed	0.90 (0.77 - 1.05)	.181
CC +AC (11)	590 (41.35)	753 (40.51)	Risk	9.12	.5206	0%	Fixed	1.06 (0.97 - 1.15)	.1965
			Odds	8.58	.5726	0%	Fixed	1.11 (0.95 - 1.29)	.2074
AA+AC (11)	1324 (92.78)	1751 (94.19)	Risk	43.53	<.0001	77.0%	Random	0.98 (0.94 - 1.02)	.2826
			Odds	20.48	.025	51.2%	Random	0.73 (0.44 - 1.20)	.2122

Table S3. Pooled meta-analysis: *MTHFR A1298C* polymorphism and risk of hypertensive disorders in pregnancy (11 studies).

Genotype (number of studies	Case N=1425 n (%)	Control N=1859 n (%)	Test of Heterogeneity Q	p	I^2(%)	Statistical Model	Test of Association Relative risk (95% CI)	p
CC (11)	101 (7.09)	108 (5.81)	19.81	.0311	49.5	Random	1.31 (0.82 – 2.07)	.2558
Caucasian (4)	37 (8.96)	72 (9.55)	3.73	.2926	19.5	Fixed	0.92 (0.62 – 1.35)	.6603
South American (1)	23(15.33)	1 (0.67)	-	-	-	-	-	-
Asian# (2)	16 (6.99)	16 (5.90)	0.10	.7565	0	Fixed	1.13 (0.58 – 2.21)	.7192
Middle East (2)	17 (7.02)	11 (4.47)	0.06	.8116	0	Fixed	1.57 (0.75 – 3.26)	.2311
African (2)	8 (2.04)	8 (1.83)	5.88	.0153	83.0	Random	2.18 (0.08 – 62.07)	.6485
AC (11)	489 (34.31)	645 (34.70)	14.87	.1367	32.8	Fixed	1.01 (0.92 – 1.11)	.7807
Caucasian (4)	190 (46.00)	344 (45.62)	5.77	.1200	48	Fixed	0.97 (0.84 – 1.11)	.6167
South American (1)	27 (18.00)	39 (26.00)	-	-	-	-	-	.2538
Asian# (2)	109 (47.60)	119 (43.91)	0.01	.9035	0	Fixed	1.05 (0.87 – 1.27)	.5987
Middle East (2)	83 (34.30)	69 (28.05)	1.96	.1619	48.9	Fixed	1.22 (0.94 – 1.58)	.1337
African (2)	80 (20.36)	74 (16.89)	2.02	.1556	50.4	Fixed	1.09 (0.82 – 1.45)	.5690
AA (11)	835 (58.60)	1106 (59.49)	7.97	.6383	0	Fixed	0.96 (0.91 – 1.02)	.1773
Caucasian (4)	186 (45.04)	338 (44.83)	2.52	.4707	0	Fixed	1.05 (0.92 – 1.20)	.4363
South American (1)	100 (66.67)	110 (73.33)	-	-	-	-	-	-
Asian# (2)	102 (44.54)	136 (50.18)	0.04	.8499	0	Fixed	0.94 (0.78 – 1.13)	.4816
Middle East (2)	142 (56.68)	166 (67.48)	0.02	.8849	0	Fixed	0.87 (0.76 – 0.99)	.0404
African (2)	305 (77.61)	356 (81.28)	0.00	.9788	0	Fixed	0.98 (0.91 – 1.05)	.5004
CC+AC	590 (41.35)	753 (40.51)	9.15	.5182	0	Fixed	1.06 (0.97 – 1.15)	.1815
AA+AC	1324 (92.78)	1751 (94.19)	42.93	<.0001	76.7	Random	0.98 (0.94 – 1.02)	.3086
**Subgroups**								
**CC Risk > 1 (7 countries)**	886 (62.18)	1290 (69.39)						
CC (8)	84 (9.46)	77 (5.97)	14.41	. 0444	51.4	Random	1.69 (0.99 – 2.88)	.0540
AC (8)	309 (34.80)	484 (37.52)	10.74	.1502	34.8	Fixed	0.95 (0.85 – 1.06)	.3563
AA (8)	493 (55.52)	729 (56.51)	7.62	.3675	8.1	Fixed	0.96 (0.89 – 1.03)	.2845
CC+AC (8)	393 (44.36)	561 (43.49)	8.98	.2541	22.1	Fixed	1.05 (0.95 – 1.16)	.3091
AA+AC (8)	802 (90.52)	1113 (86.28)	17.52	.0143	60.1	Random	0.95 (0.92 – 0.99)	.0258
**CC risk < 1 (3 countries)**	539 (37.82)	569 (30.61)						
CC (3)	17 (3.15)	31 (5.45)	2.26	.3232	11.5	Fixed	0.55 (0.29 – 1.02)	.0569
AC (3)	180 (33.40)	161 (28.30)	0.37	.8308	0	Fixed	1.17 (0.98 – 1.39)	.0769
AA(3)	342 (63.45)	377 (66.26)	0.32	.8529	0	Fixed	0.96 (0.89 – 1.05)	.3779
CC+AC (3)	197 (36.55)	192 (33.74)	0.04	.9826	0	Fixed	1.07 (0.92 – 1.25)	.3789
AA+AC (3)	522 (96.85)	538 (94.55)	6.29	.0431	68.2	Random	1.04 (0.99 – 1.10)	.1502

Note:
1. #: Asia include East and South Asia
2. CC risk > 1 (7 countries): Netherland (2 studies), Ecuador, China, Sri Lanka, Iran, Turkey, and Tunisia
3. CC risk < 1 (3 countries): Australia, Spain, and South Africa

Table S4a. Pooled meta-analysis: *MTHFR C677T polymorphism* and risks of hypertensive disorders in pregnancy (HDP) per HDP types. (71 studies)

Genotype (number of studies)	Case N= 8064 n (%)	Control N= 13232 n (%)	Test of Heterogeneity Q	p	I^2 (%)	Statistical Model	Test of Association Relative risk ratio (95% CI)	p
**TT (71)**	1087 (13.48)	1410 (10.66)	107.30	.0028	34.8	Random	1.28 (1.15 – 1.43)	<.0001
**PE (57)**	799 (13.61)	1221 (10.58)	94.98	.0009	41.0	Random	1.29 (1.13 – 1.47)	.0001
Caucasian (25)	330 (11.71)	669 (9.75)	25.02	.4046	4.1	Fixed	1.15 (1.00 – 1.32)	.0473
Hispanic (6)	177 (30.68)	282 (30.62)	9.09	.1057	45.0	Fixed	0.98 (0.83 – 1.15)	.7838
South American (4)	62 (16.40)	66 (11.89)	1.47	.6891	0	Fixed	1.40 (1.01 – 1.93)	.0405
East Asian (8)	156 (29.38)	139 (11.08)	10.89	.1433	35.7	Fixed	2.06 (1.68 – 2.53)	<.0001
South Asian (4)	15 (2.67)	31 (3.13)	0.73	.8654	0	Fixed	0.94 (0.49 – 1.81)	.8606
Middle East (6)	40 (6.21)	34 (6.46)	6.35	.2734	21.3	Fixed	0.92 (0.58 – 1.46)	.7254
African (4)	19 (5.22)	0	1.92	.5901	0	Fixed	11.38 (2.73 – 47.55)	.0009
**GH (4)**	80 (23.81)	47 (14.37)	2.44	.4872	0	Fixed	1.65 (1.20 – 2.29)	.0023
East Asian (3)	66 (27.97)	35 (15.56)	1.38	.5015	0	Fixed	1.81 (1.26 – 2.60)	.0014
Middle East (1)	14 (14.00)	12 (11.76)	-	-	-	-	-	-
**Mix (10)**	208 (11.21)	142 (10.44)	5.00	.8344	0	Fixed	1.08 (0.87 – 1.33)	.4837
Caucasian (2)	95 (11.45)	31 (11.15)	0.22	.6412	0	Fixed	1.11 (0.75 – 1.65)	.5927
Hispanic (1)	38 (20.21)	43 (22.16)	-	-	-	-	-	-
East Asian (6)	74 (15.16)	66 (12.00)	3.21	.6683	0	Fixed	1.20 (0.86 – 1.68)	.2845
African (1)	1 (0.29)	2 (0.59)	-	-	-	-	-	-
**CT (71)**	3142 (38.96)	5166 (39.04)	103.13	.0049	33.1	Random	1.01 (0.96 – 1.06)	.7256
**PE (57)**	2229 (37.95)	4575 (39.63)	61.38	.258	10.4	Fixed	0.98 (0.93 – 1.02)	.2461
Caucasian (25)	1159 (41.13)	2889 (42.11)	19.57	.6674	0	Fixed	1.01 (0.95 – 1.07)	.8459
Hispanic (6)	269 (46.62)	427 (46.36)	2.53	.7712	0	Fixed	1.01 (0.91 – 1.13)	.8015
South American (4)	173 (45.77)	258 (46.49)	3.24	.3558	7.5	Fixed	0.94 (0.81 – 1.08)	.3468
East Asian (8)	234 (44.07)	531 (42.31)	10.00	.1887	30.0	Fixed	0.88 (0.78 – 0.99)	.0354
South Asian (4)	94 (16.76)	199 (20.08)	7.74	.0516	61.3	Fixed	0.77 (0.61 – 0.98)	.0335
Middle East (6)	214 (33.23)	159 (30.23)	5.59	.3481	10.6	Fixed	1.05 (0.88 – 1.24)	.5985
African (4)	86 (23.63)	112 (25.63)	4.63	.2006	35.3	Fixed	1.00 (0.79 – 1.26)	.9787
**GH (4)**	156 (46.43)	153 (46.79)	11.79	.0081	74.6	Random	0.99 (0.70 – 1.41)	.9751
East Asian (3)	103 (43.64)	106 (47.11)	10.23	.006	80.4	Random	0.94 (0.57 – 1.56)	.8131
Middle East (1)	53 (53.00)	47 (46.08)	-	-	-	-	-	-
**Mix (10)**	757 (40.81)	438 (32.21)	20.39	.0157	55.9	Random	1.22 (1.04 – 1.43)	.0162
Caucasian (2)	405 (48.80)	112 (40.29)	0.06	.8129	0	Fixed	1.20 (1.02 – 1.42)	.0281
Hispanic (1)	91 (48.80)	97 (50.00)	-	-	-	-	-	-
East Asian (6)	211 (43.24)	191 (34.73)	16.19	.0063	69.1	Random	1.35 (0.98 – 1.86)	.0621
African (1)	50 (14.33)	38 (11.24)	-	-	-	-	-	-
**CC (71)**	3835 (47.56)	6656 (50.30)	120.18	.0002	41.8	Random	0.94 (0.90 – 0.98)	.0017
**PE (57)**	2845 (48.44)	5749 (49.80)	72.50	.0682	22.8	Fixed	0.96 (0.93 – 0.99)	.0313
Caucasian (25)	1329 (47.16)	3302 (48.13)	18.46	.7803	0	Fixed	0.96 (0.91 – 1.02)	.1639
Hispanic (6)	131 (22.70)	212 (23.02)	5.66	.3409	11.6	Fixed	1.00 (0.83 – 1.21)	.996
South American (4)	143 (37.83)	231 (41.62)	4.85	.1833	38.1	Fixed	0.96 (0.81 – 1.13)	.6263
East Asian (8)	141 (26.55)	585 (46.61)	7.39	.3897	5.2	Fixed	0.76 (0.65 – 0.88)	.0004
South Asian (4)	452 (80.57)	761 (76.79)	9.64	.0219	68.9	Random	1.06 (0.95 – 1.17)	.3296
Middle East (6)	390 (60.56)	333 (63.31)	5.29	.3818	5.4	Fixed	0.99 (0.90 – 1.08)	.7435
African (4)	259 (71.15)	325 (74.37)	4.99	.1724	39.9	Fixed	0.93 (0.86 – 1.00)	.0864
**GH (4)**	100 (29.76)	127 (38.84)	6.67	.0831	55.0	Fixed	0.76 (0.62 – 0.94)	.0106
East Asian (3)	67 (28.39)	84 (37.33)	6.70	.0351	70.1	Random	0.80 (0.50 – 1.29)	.3628
Middle East (1)	33 (33.00)	43 (42.16)	-	-	-	-	-	-
**Mix (10)**	890 (47.98)	780 (57.35)	33.60	.0001	73.2	Random	0.83 (0.71 – 0.96)	.0102
Caucasian (2)	330 (39.76)	135 (48.56)	0.03	.8672	0	Fixed	0.81 (0.69 – 0.94)	.0062
Hispanic (1)	59 (31.38)	54 (27.84)	-	-	-	-	-	-
East Asian (6)	203 (41.60)	293 (53.27)	15.01	.0103	66.%	Random	0.75 (0.59 – 0.95)	.0159
African (1)	298 (85.39)	298 (88.17)	-	-	-	-	-	-
**TT+CT (71)**	4229 (52.44)	6576 (49.70)	106.46	.0033	34.2	Random	1.07 (1.03 – 1.11)	.0002

**PE (57)**	2845 (48.44)	5749 (49.80)	67.96	.1313	17.6	Fixed	1.04 (1.00 – 1.07)	.0287
Caucasian (25)	1489 (52.84)	3558 (51.87)	18.67	.7694	0	Fixed	1.03 (0.99 – 1.08)	.1559
Hispanic (6)	446 (77.30)	709 (76.98)	5.78	.3285	13.5	Fixed	1.00 (0.94 – 1.06)	.9961
South American (4)	235 (62.17)	324 (58.38)	5.38	.146	44.2	Fixed	1.03 (0.93 – 1.14)	.6174
East Asian (8)	390 (73.45)	670 (53.39)	6.35	.4993	0	Fixed	1.15 (1.07 – 1.23)	.0001
South Asian (4)	109 (19.43)	230 (23.21)	7.93	.0475	62.2	Random	0.83 (0.56 – 1.22)	.3382
Middle East (6)	254 (39.44)	193 (36.69)	4.96	.4211	0	Fixed	1.02 (0.89 – 1.19)	.743
African (4)	105 (28.85)	112 (25.63)	1.56	.6695	0	Fixed	1.20 (0.98 – 1.48)	.0801
**GH (4)**	236 (70.24)	200 (61.16)	8.35	.0393	64.1	Random	1.14 (0.94 – 1.38)	.1749
East Asian (3)	169 (71.61)	141 (62.67)	8.30	.0158	75.9	Random	1.14 (0.85 – 1.52)	.3868
Middle East (1)	67 (67.00)	59 (57.84)	-	-	-	-	-	-
**Mix (10)**	965 (52.02)	580 (42.65)	27.82	.001	67.6	Random	1.21 (1.05 – 1.26)	<.0001
Caucasian (2)	500 (60.24)	143 (51.44)	0.00	.9652	0	Fixed	1.18 (1.04 – 1.35)	.0116
Hispanic (1)	129 (68.62)	140 (72.16)	-	-	-	-	-	-
East Asian (6)	285 (58.40)	257 (46.73)	18.19	.0027	72.5	Random	1.34 (1.04 – 1.72)	.0251
African (1)	51 (14.61)	40 (11.83)	-	-	-	-	-	-
**CC+CT (71)**	6977 (86.52)	11822 (89.34)	185.18	<.0001	62.3	Random	0.98 (0.96 – 0.99)	.0023
**PE (57)**	5074 (86.40)	10324 (89.42)	128.50	<.0001	56.4	Random	0.97 (0.96 – 0.98)	<.0001
Caucasian (25)	2488 (88.29)	6191 (90.25)	24.83	.415	3.3	Fixed	0.98 (0.97 – 1.00)	.0552
Hispanic (6)	400 (69.32)	639 (69.38)	9.92	.0776	49.6	Fixed	1.01 (0.94 – 1.08)	.7806
South American (4)	316 (83.60)	489 (88.11)	2.25	.5215	0	Fixed	0.95 (0.90 – 0.99)	.0475
East Asian (8)	375 (70.62)	1116 (88.92)	5.61	.5854	0	Fixed	0.83 (0.78 – 0.88)	<.0001
South Asian (4)	546 (97.33)	960 (96.87)	0.78	.8549	0	Fixed	1.00 (0.98 – 1.02)	.8334
Middle East (6)	604 (93.79)	492 (93.54)	8.34	.1383	40.1	Fixed	1.01 (0.98 – 1.04)	.7176
African (4)	345 (94.78)	437 (100.0)	56.03	<.0001	94.6	Random	0.94 (0.87 – 1.02)	.1506
**GH (4)**	256 (29.76)	280 (85.63)	7.27	.0635	58.8	Fixed	0.89 (0.83 – 0.96)	.002
East Asian (3)	170 (76.19)	190 (84.44)	4.66	.0975	57.0	Fixed	0.85 (0.77 – 0.94)	.0012
Middle East (1)	86 (86.00)	90 (88.24)	-	-	-	-	-	-
**Mix (10)**	1647 (88.79)	1218 (89.56)	10.35	.3229	13.0	Fixed	0.99 (0.97 – 1.02)	.4767
Caucasian (2)	735 (88.55)	247 (88.85)	0.30	.5846	0	Fixed	0.99 (0.94 – 1.04)	.5888
Hispanic (1)	150 (79.79)	151 (77.84)	-	-	-	-	-	-
East Asian (6)	414 (84.84)	484 (88.00)	3.37	.6436	0	Fixed	0.97 (0.93 – 1.02)	.2698
African (1)	348 (99.71)	336 (99.41)	-	-	-	-	-	-

PE: pre-eclampsia, eclampsia and HELLP syndrome; GH: gestational hypertension; Mix: combined PE and GH

Table S4b. Pooled meta-analysis: *MTHFR A1298C polymorphism* and risk of hypertensive disorders in pregnancy (HDP) per HDP types. (11 studies)

Genotype (number of studies)		Case N=1425 n (%)	Control N=1859 n (%)	Test of Heterogeneity Q	*p*	I^2	Statistical Model	Test of Association Relative risk ratio (95% CI)	*p*
**CC (11)**		101 (7.09)	108(5.81)	19.83	.0309	49.6%	Random	1.30 (0.82 – 2.07)	.2586
**PE (9)**		94 (9.18)	96 (6.76)	17.61	.0244	54.6%	Random	1.45 (0.87 – 2.45)	.1576
	Caucasian (4)	37 (8.96)	72 (9.55)	3.73	.2926	19.5%	Fixed	0.92 (0.62 – 1.35)	.6603
	South American (1)	23 (15.33)	1 (0.67)	-	-	-	-	-	-
	South Asian (1)	13 (7.51)	12 (7.02)	-	-	-	-	-	-
	Middle East (2)	17 (7.02)	11 (4.47)	0.06	.8116	0%	Fixed	1.57 (0.75 – 3.26)	.2311
	African (1)	4 (9.09)	0 (0.00)	-	-	-	-	-	-
**GH**	East Asian (1)	3(5.56)	4 (4.00)	-	-	-	-	-	-
**Mix**	African (1)	4 (1.18)	8 (2.37)	-	-	-	-	-	-
**AC (11)**		489 (34.31)	645 (34.70)	14.81	.1393	32.5%	Fixed	1.01 (0.92 – 1.11)	.8107
**PE (9)**		389 (37.99)	542 (38.14)	14.01	.0816	42.9%	Fixed	0.99 (0.89 – 1.10)	.8137
	Caucasian (4)	190 (46.00)	344 (45.62)	5.77	.1232	48.0%	Fixed	0.97 (0.84 – 1.11)	.6167
	South American (1)	27 (18.00)	39 (26.00)	-	-	-	-	-	-
	South Asian (1)	89 (51.45)	83 (48.54)	-	-	-	-	-	-
	Middle East (2)	83 (34.30)	69 (28.05)	1.96	.1619	48.9%	Fixed	1.22 (0.94 – 1.58)	.1337
	African (1)	0 (0.00)	7 (7.00)	-	-	-	-	-	-
**GH**	East Asian (1)	20 (37.04)	36 (36.00)	-	-	-	-	-	-
**Mix**	African (1)	80 (22.92)	67 (19.82)	-	-	-	-	-	-
**AA (11)**		835 (58.60)	1106 (59.49)	7.97	.6321	0%	Fixed	0.96 (0.91 – 1.02)	.1671
**PE (9)**		539 (52.64)	783 (55.10)	7.77	.4564	0%	Fixed	0.96 (0.89 – 1.03)	.225
	Caucasian (4)	186 (45.04)	338 (44.83)	2.53	.4707	0%	Fixed	1.05 (0.92 – 1.20)	.4363
	South American (1)	100 (66.76)	110 (73.33)	-	-	-	-	-	-
	South Asian (1)	71 (41.04)	76 (44.44)	-	-	-	-	-	-
	Middle East (2)	142 (58.68)	166 (67.48)	0.02	.8849	0%	Fixed	0.87 (0.76 – 0.99)	.0404
	African (1)	40 (90.91)	93 (93.00)	-	-	-	-	-	-
**GH**	East Asian (1)	31 (57.40)	60 (60.00)	-	-	-	-	-	-
**Mix**	African (1)	265 (75.93)	263 (77.81)	-	-	-	-	-	-
**CC+AC (11)**		590 (41.35)	753 (40.51)	9.12	.5206	0%	Fixed	1.06 (0.97 – 1.15)	.1965
**PE (9)**		483 (47.26)	638 (44.90)	9.04	.3388	11.5%	Fixed	1.05 (0.97 – 1.15)	.2459
	Caucasian (4)	227 (54.96)	416 (55.17)	2.66	.4478	0%	Fixed	0.96 (0.85 – 1.07)	.4504
	South American (1)	50 (33.33)	40 (26.67)	-	-	-	-	-	-
	South Asian (1)	102 (58.96)	95 (55.56)	-	-	-	-	-	-
	Middle East (2)	100 (41.32)	80 (32.52)	1.55	.2135	0%	Fixed	1.27 (1.01 – 1.59)	.0403
	African (1)	4 (9.09)	7 (7.00)	-	-	-	-	-	-
**GH**	East Asian (1)	23 (42.59)	40 (40.00)	-	-	-	-	-	-
**Mix**	African (1)	84 (24.07)	75 (22.19)	-	-	-	-	-	-
**AA+AC (11)**		1324 (92.78)	1751 (94.19)	43.53	<.0001	77.0%	Random	0.98 (0.94 – 1.02)	.2826
**PE (9)**		928 (90.80)	1325 (93.24)	31.88	<.0001	74.9%	Random	0.97 (0.93 – 1.02)	.2493
	Caucasian (4)	376 (91.04)	682 (90.45)	7.07	.0695	57.6%	Fixed	1.01 (0.97 – 1.05)	.6944
	South American (1)	127 (84.67)	149 (99.33)	-	-	-	-	-	-
	South Asian (1)	160 (92.49)	159 (92.98)	-	-	-	-	-	-
	Middle East (2)	225 (92.98)	235 (95.53)	0.53	.4669	0%	Fixed	0.97 (0.93 – 1.02)	.2279
	African (1)	40 (90.91)	100 (100)	-	-	-	-	-	-
**GH**	East Asian (1)	51 (94.44)	96 (96.00)	-	-	-	-	-	-
**Mix**	African (1)	345 (98.85)	330 (97.63)	-	-	-	-	-	-

PE: pre-eclampsia, eclampsia and HELLP syndrome; GH: gestational hypertension; Mix: combined PE and GH

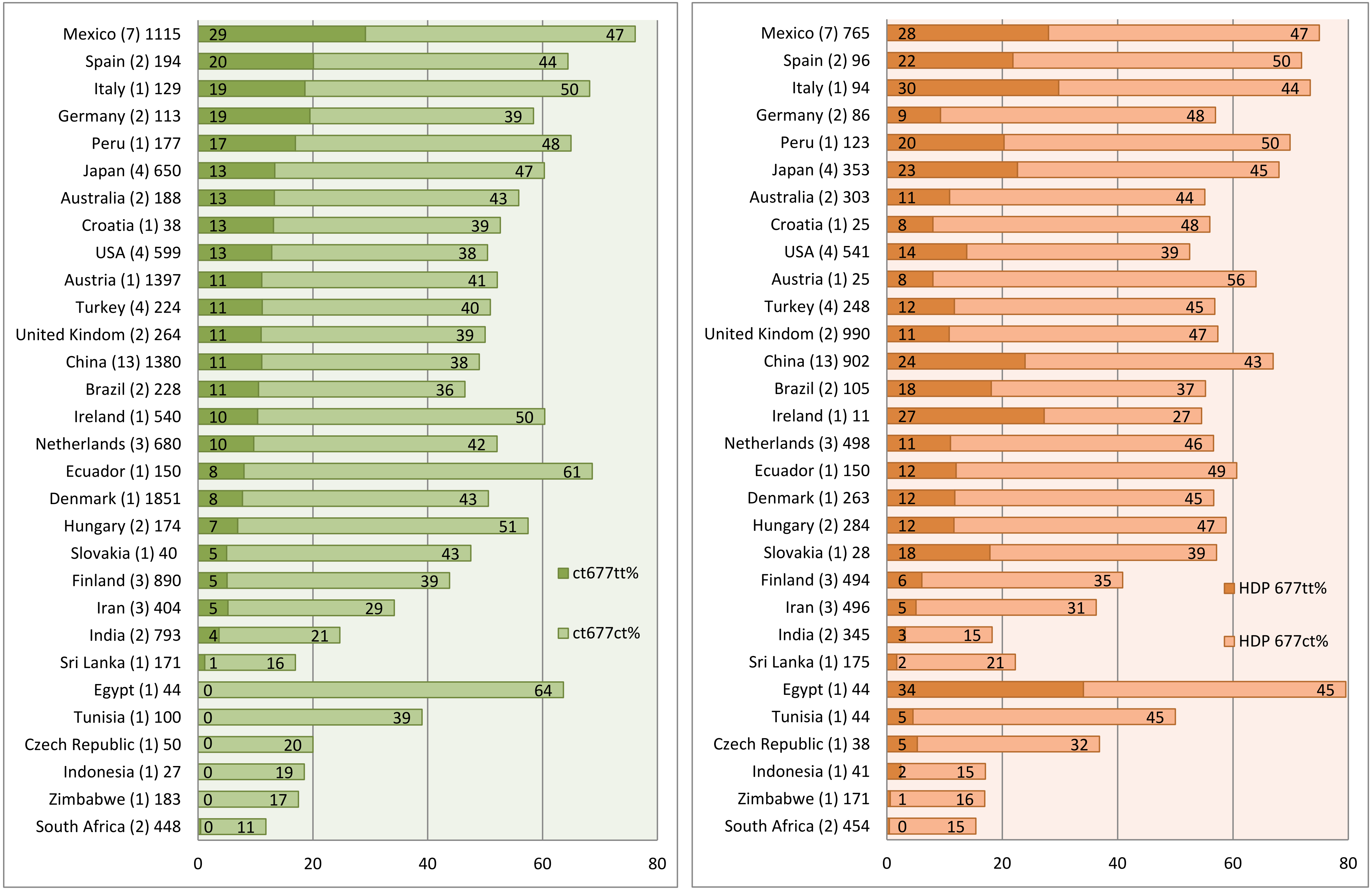

Figure S1a. The percentage of *MTHFR* 677 CT and TT polymorphisms per control and hypertensive disorders in pregnancy (HDP) case groups.

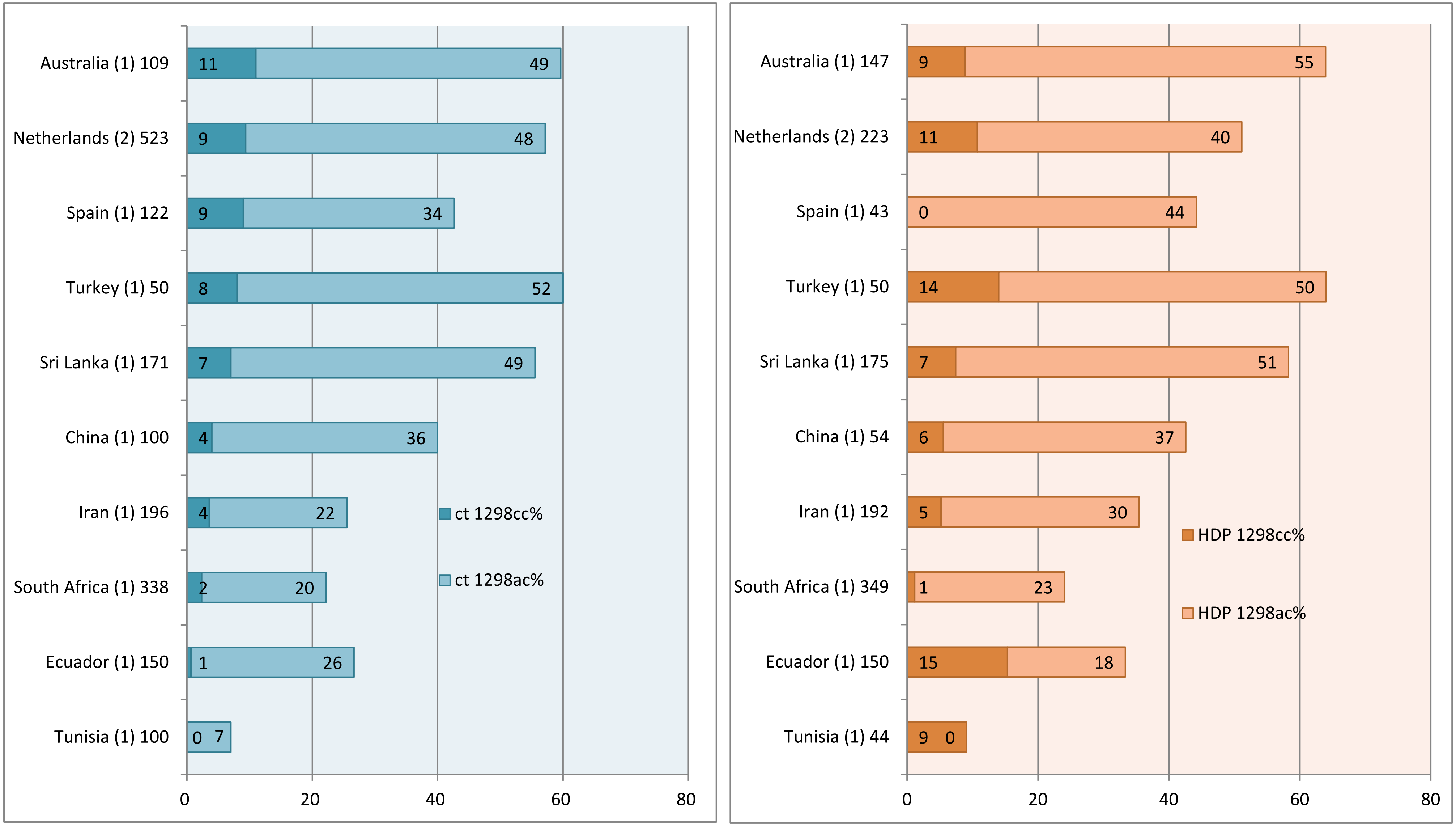

Figure S1b. The percentage of *MTHFR* 1298 AC and CC polymorphisms per control and hypertensive disorders in pregnancy (HDP) case groups.

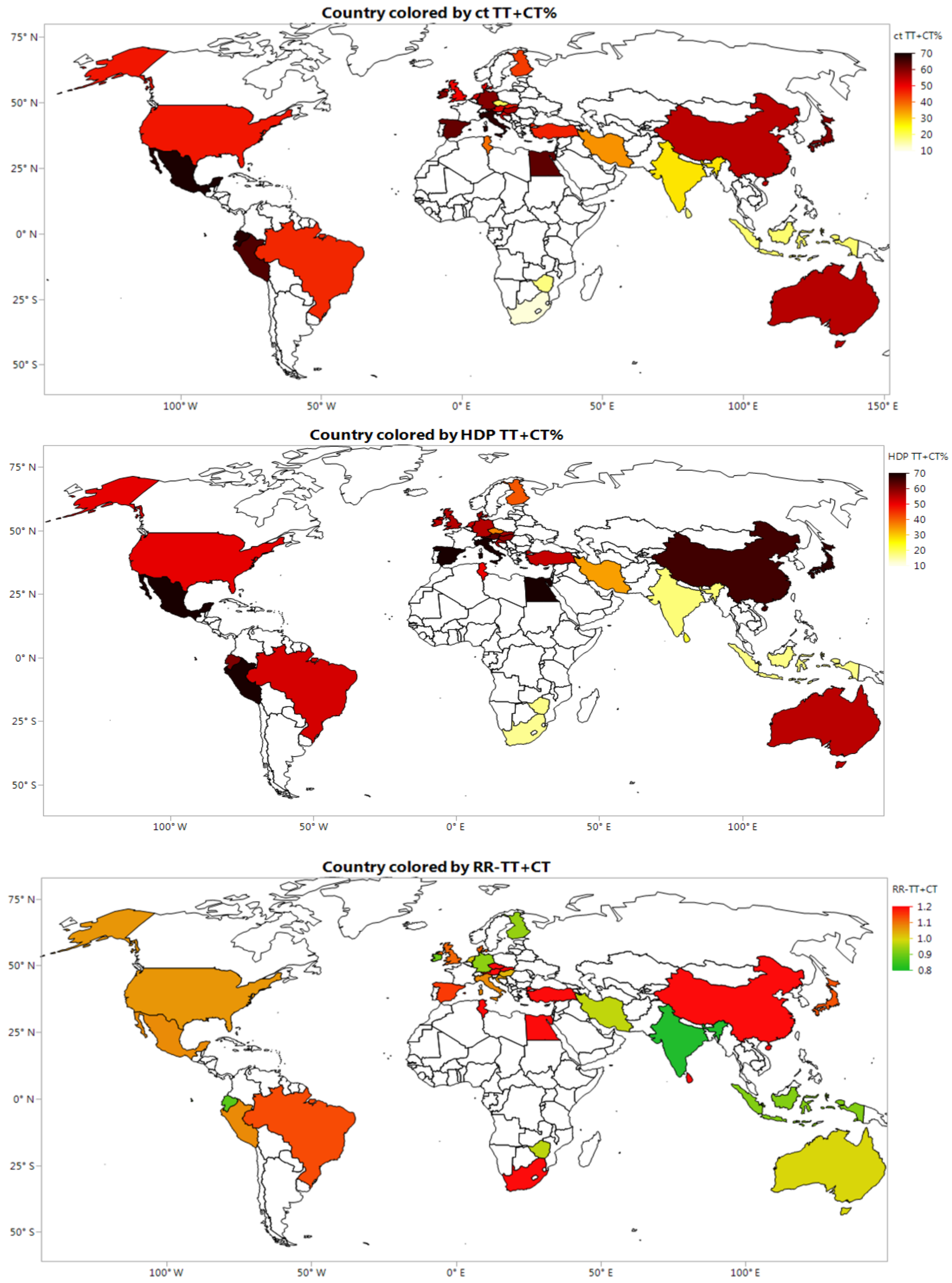

Figure S2a. Geographic information maps for % *MTHFR 677 TT plus CT* polymorphism per control and hypertensive disorders in pregnancy (HDP) groups, and HDP risks.

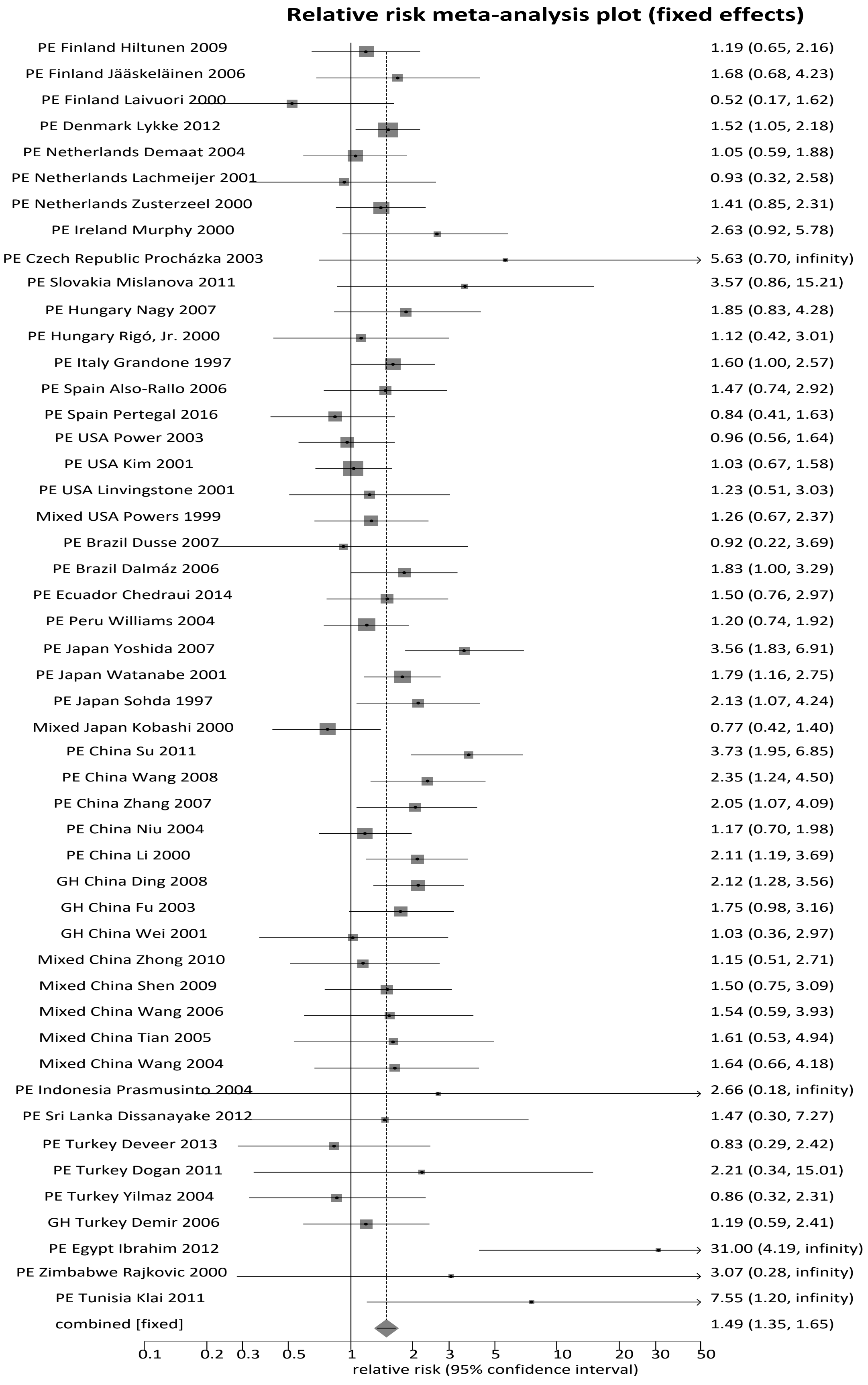

Figure S3a. Forest plot for *MTHFR C677T with* types of hypertensive disorders in pregnancy for countries of *TT* risk > 1.

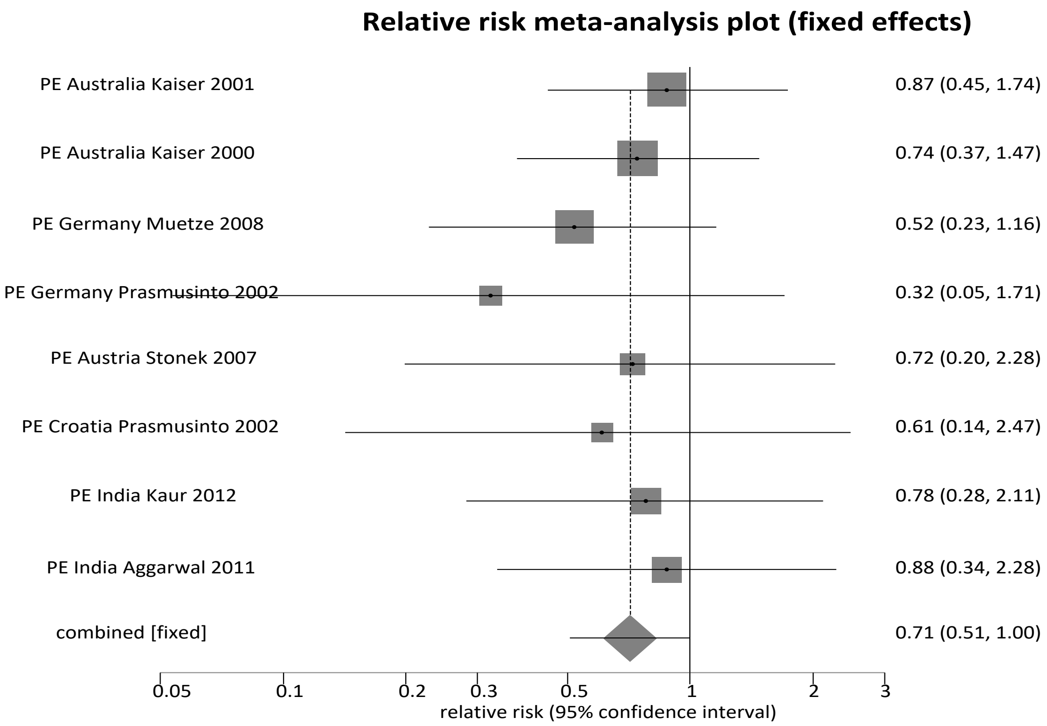

Figure S3b. Forest plot for *MTHFR C677T* with types of hypertensive disorders in pregnancy for countries of *TT* risk < 1.

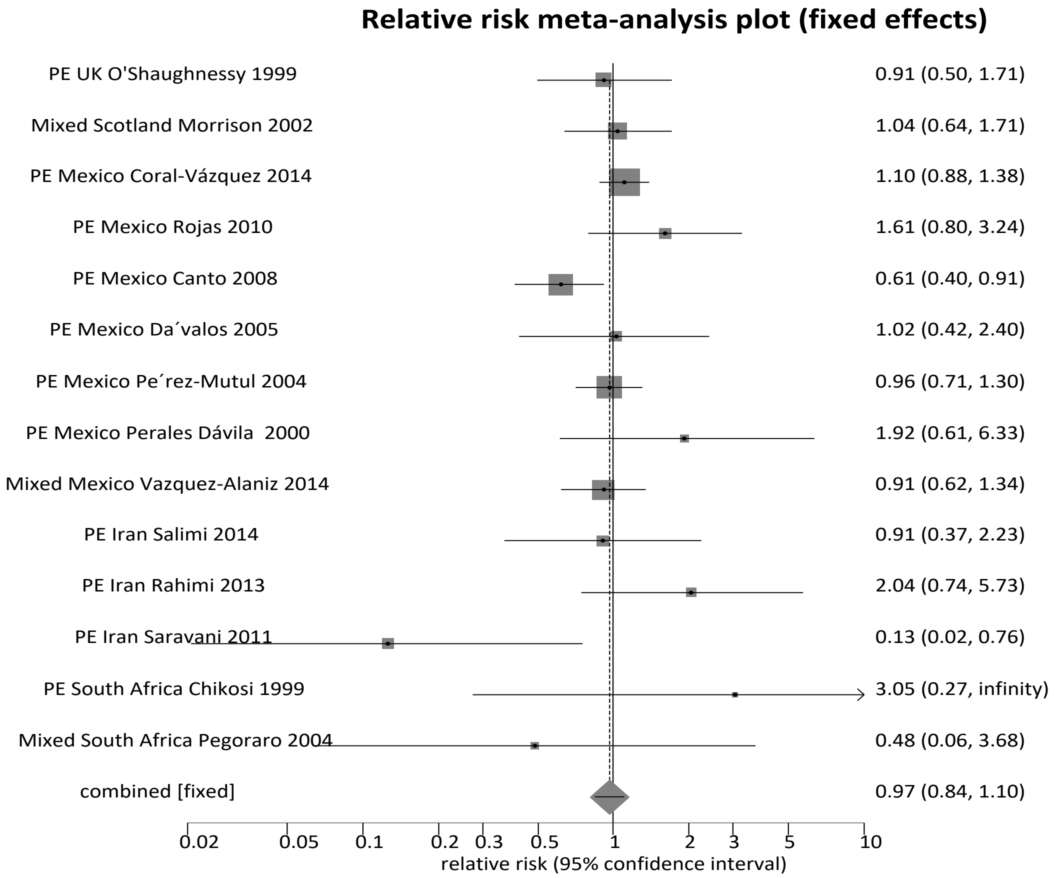

Figure S3c. Forest plot for *MTHFR C677T with* types of hypertensive disorders in pregnancy for countries of *TT* risk ~ 1.

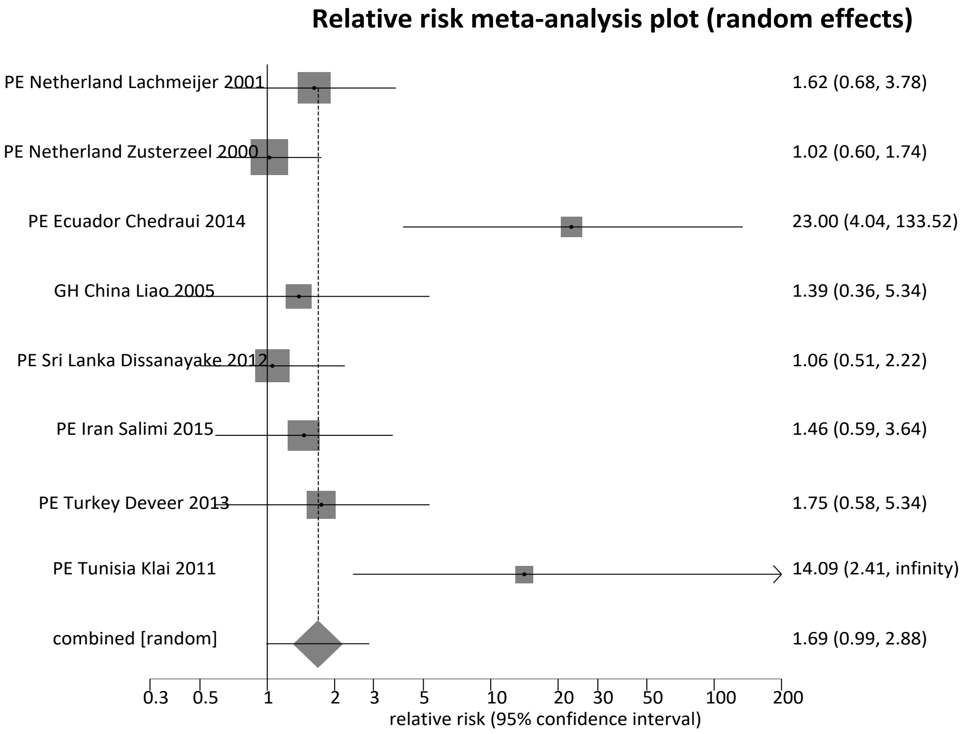

Figure S4a. Forest plot for *MTHFR A1298C with* types of hypertensive disorders in pregnancy for countries of *CC* risk > 1.

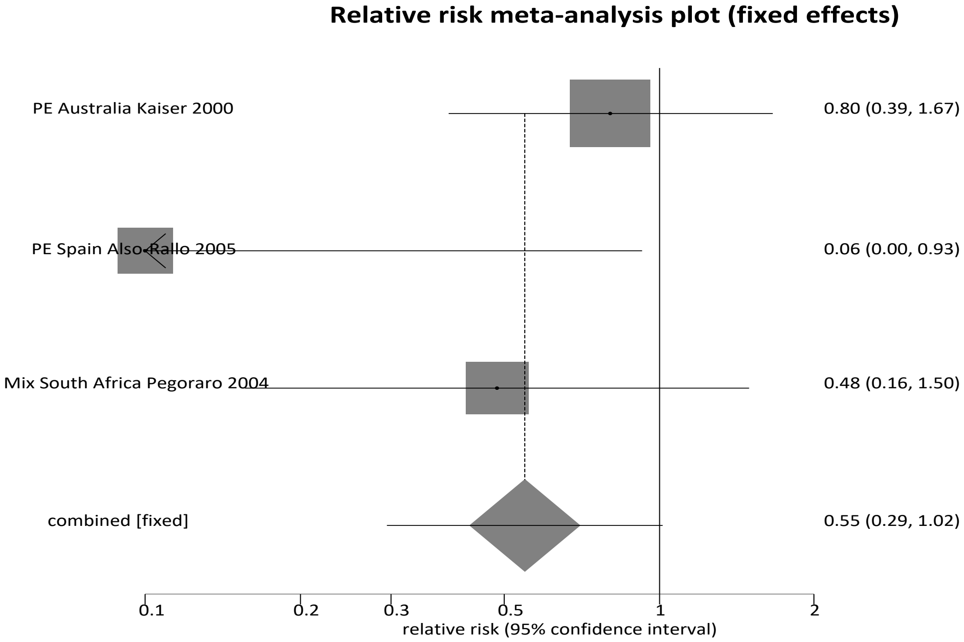

Figure S4b. Forest plot for *MTHFR A1298C with* types of hypertensive disorders in pregnancy for countries of *CC* risk < 1.

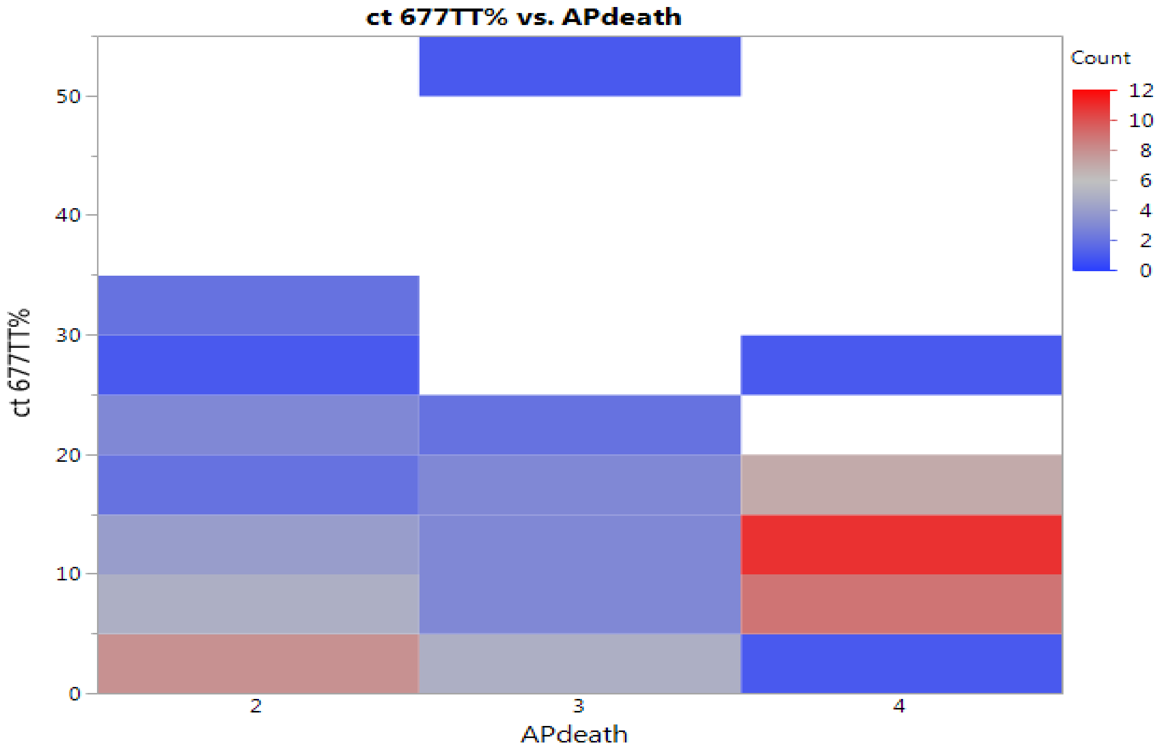

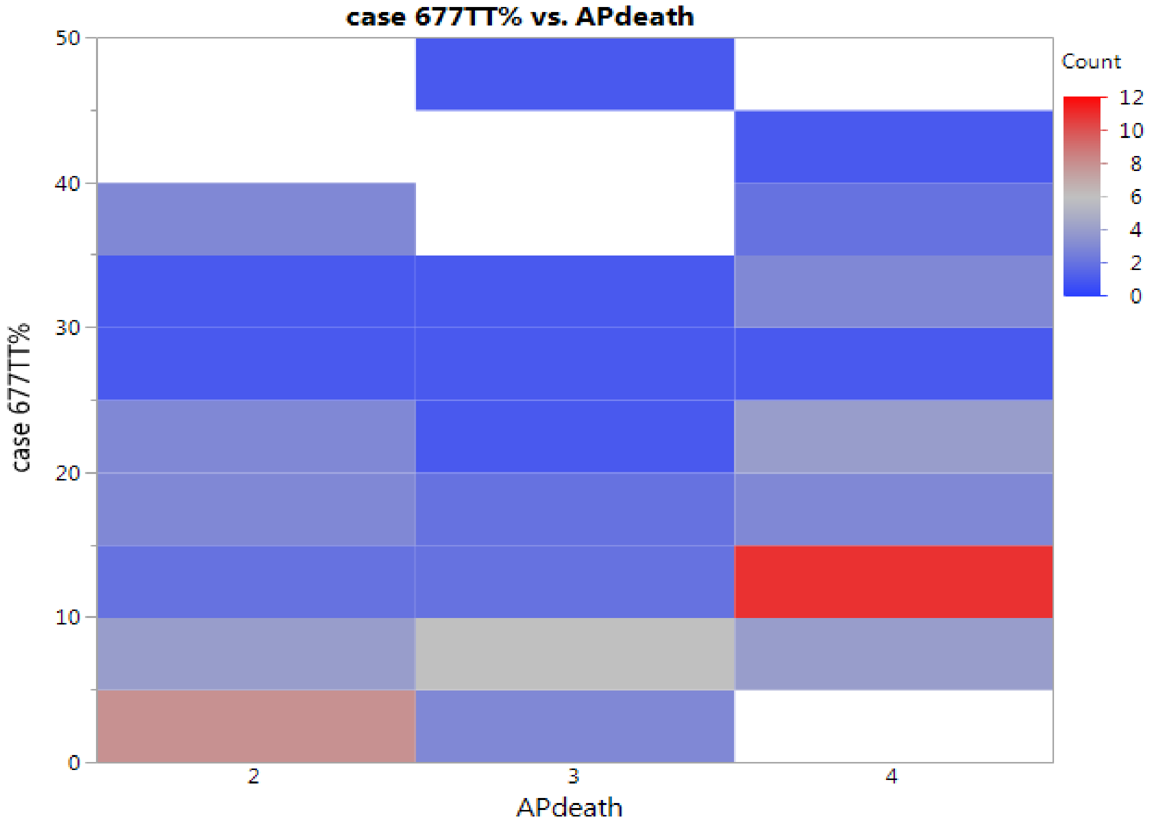

Figure S5a. Heat maps of *MTHFR 677* homozygous *TT* genotypes for control and case groups in association with the rate of deaths from air pollution.

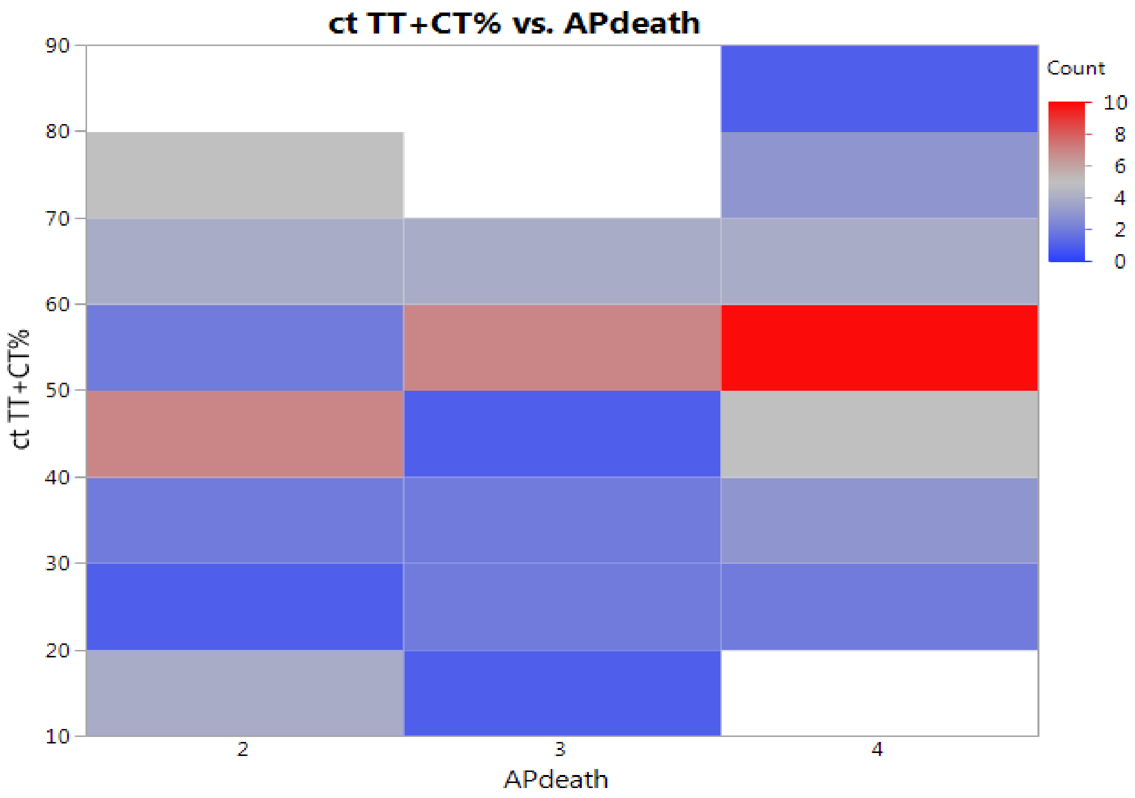

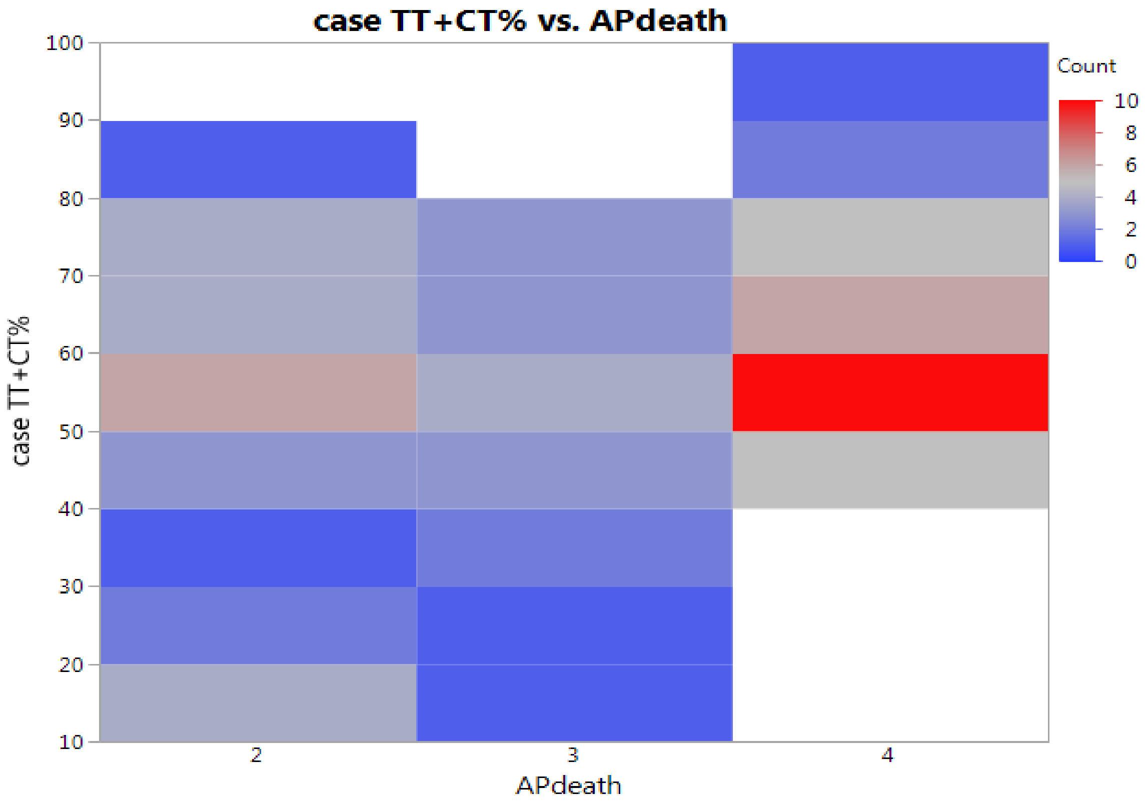

Figure S5b. Heat maps of *MTHFR 677 TT plus CT* genotypes for control and case groups in association with the rate of deaths from air pollution.
